# Computer-Aided Imaging Analysis of Probe-Based Confocal Laser Endomicroscopy With Molecular Labeling and Gene Expression Identifies Markers of Response to Biological Therapy in IBD Patients: The Endo-Omics Study

**DOI:** 10.1093/ibd/izac233

**Published:** 2022-11-15

**Authors:** Marietta Iacucci, Louisa Jeffery, Animesh Acharjee, Enrico Grisan, Andrea Buda, Olga M Nardone, Samuel C L Smith, Nunzia Labarile, Davide Zardo, Bella Ungar, Stuart Hunter, Ren Mao, Rosanna Cannatelli, Uday N Shivaji, Tommaso Lorenzo Parigi, Gary M Reynolds, Georgios V Gkoutos, Subrata Ghosh

**Affiliations:** National Institute for Health Research Birmingham Biomedical Research Centre, University of Birmingham, Birmingham, UK; Gastroenterology, Queen Elizabeth Hospital, University Hospitals Birmingham NHS Foundation Trust, Birmingham, UK; Institute of Immunology and Immunotherapy, University of Birmingham, Birmingham, UK; Gastroenterology, Queen Elizabeth Hospital, University Hospitals Birmingham NHS Foundation Trust, Birmingham, UK; Institute of Immunology and Immunotherapy, University of Birmingham, Birmingham, UK; Institute of Cancer and Genomic Sciences, University of Birmingham, Birmingham, UK; National Institute for Health Research Surgical Reconstruction, Queen Elizabeth Hospital Birmingham, Birmingham, UK; Department of Information Engineering, University of Padova, Padova, Italy; School of Engineering Computer Science and Informatics, London South Bank University, London, UK; Gastroenterology Unit, Department of Gastrointestinal Oncological Surgery, S. Maria del Prato Hospital, Feltre, Italy; Institute of Immunology and Immunotherapy, University of Birmingham, Birmingham, UK; Institute of Immunology and Immunotherapy, University of Birmingham, Birmingham, UK; Institute of Immunology and Immunotherapy, University of Birmingham, Birmingham, UK; Gastroenterology, Queen Elizabeth Hospital, University Hospitals Birmingham NHS Foundation Trust, Birmingham, UK; Institute of Immunology and Immunotherapy, University of Birmingham, Birmingham, UK; Institute of Immunology and Immunotherapy, University of Birmingham, Birmingham, UK; Department of Gastroenterology, The First Affiliated Hospital of Sun Yat-Sen University, Guangzhou, China; Institute of Immunology and Immunotherapy, University of Birmingham, Birmingham, UK; Gastroenterology, Queen Elizabeth Hospital, University Hospitals Birmingham NHS Foundation Trust, Birmingham, UK; Institute of Immunology and Immunotherapy, University of Birmingham, Birmingham, UK; Institute of Immunology and Immunotherapy, University of Birmingham, Birmingham, UK; Institute of Cancer and Genomic Sciences, University of Birmingham, Birmingham, UK; National Institute for Health Research Birmingham Biomedical Research Centre, University of Birmingham, Birmingham, UK; Institute of Immunology and Immunotherapy, University of Birmingham, Birmingham, UK; Institute of Cancer and Genomic Sciences, University of Birmingham, Birmingham, UK; National Institute for Health Research Birmingham Biomedical Research Centre, University of Birmingham, Birmingham, UK; Gastroenterology, Queen Elizabeth Hospital, University Hospitals Birmingham NHS Foundation Trust, Birmingham, UK; Institute of Immunology and Immunotherapy, University of Birmingham, Birmingham, UK; APC Microbiome Ireland, College of Medicine and Health, University College Cork, Cork, Ireland

**Keywords:** ulcerative colitis, Crohn’s disease, biological agents, probe confocal laser endomicroscopy, RNA transcriptomics, endoscopic molecular labeling, artificial intelligence

## Abstract

**Background:**

We aimed to predict response to biologics in inflammatory bowel disease (IBD) using computerized image analysis of probe confocal laser endomicroscopy (pCLE) in vivo and assess the binding of fluorescent-labeled biologics ex vivo. Additionally, we investigated genes predictive of anti-tumor necrosis factor (TNF) response.

**Methods:**

Twenty-nine patients (15 with Crohn’s disease [CD], 14 with ulcerative colitis [UC]) underwent colonoscopy with pCLE before and 12 to 14 weeks after starting anti-TNF or anti-integrin α4β7 therapy. Biopsies were taken for fluorescein isothiocyanate–labeled infliximab and vedolizumab staining and gene expression analysis. Computer-aided quantitative image analysis of pCLE was performed. Differentially expressed genes predictive of response were determined and validated in a public cohort.

**Results:**

In vivo, vessel tortuosity, crypt morphology, and fluorescein leakage predicted response in UC (area under the receiver-operating characteristic curve [AUROC], 0.93; accuracy 85%, positive predictive value [PPV] 89%; negative predictive value [NPV] 75%) and CD (AUROC, 0.79; accuracy 80%; PPV 75%; NPV 83%) patients. Ex vivo, increased binding of labeled biologic at baseline predicted response in UC (UC) (AUROC, 83%; accuracy 77%; PPV 89%; NPV 50%) but not in Crohn’s disease (AUROC 58%). A total of 325 differentially expressed genes distinguished responders from nonresponders, 86 of which fell within the most enriched pathways. A panel including ACTN1, CXCL6, LAMA4, EMILIN1, CRIP2, CXCL13, and MAPKAPK2 showed good prediction of anti-TNF response (AUROC >0.7).

**Conclusions:**

Higher mucosal binding of the drug target is associated with response to therapy in UC. In vivo, mucosal and microvascular changes detected by pCLE are associated with response to biologics in inflammatory bowel disease. Anti-TNF–responsive UC patients have a less inflamed and fibrotic state pretreatment. Chemotactic pathways involving CXCL6 or CXCL13 may be novel targets for therapy in nonresponders.

## Teaser

Confocal laser endomicroscopy, both in vivo and ex vivo, can predict response to anti-tumor necrosis factor and anti-integrin therapy in inflammatory bowel disease. Gene expression analysis identified differences between responders and nonresponders

## Graphical Abstract

**Figure:**
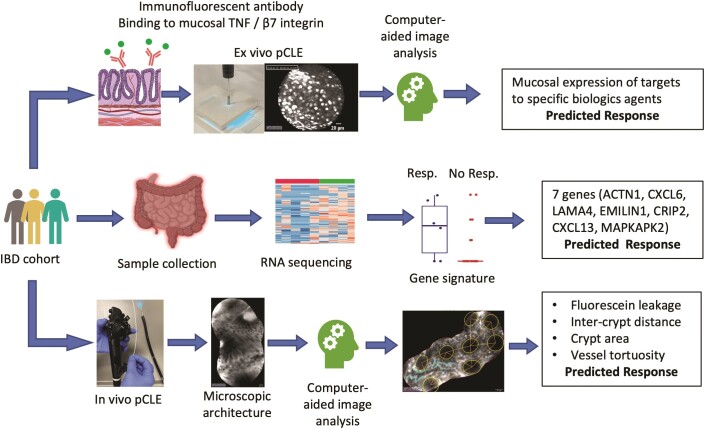
Overview of the study methodology.

**Key Messages**

**What is already known?** A significant percentage of inflammatory bowel disease patients do not respond to biologic therapy, and predictors of response are needed.

**What is new here?** Microscopic alterations and mucosal binding of drugs assessed by confocal laser endomicroscopy can predict response. Gene differentially expressed in responders and nonresponders were found.

**How can this study help patient care?** Our predictive markers can improve choice of treatment, reducing time to achieve disease control while sparing side effects and costs of ineffective therapy. Gene analysis can identify novel targets for therapy.

## Introduction

Over the past decade, several new biologics and small molecule agents have been licensed for use in inflammatory bowel disease (IBD), and many more are expected in the coming years. However, despite the increase in treatment options, response rates remain modest, with primary nonresponse occurring in around 35% to 40% of those treated with anti-tumor necrosis factor (TNF) therapy,^1^ vedolizumab,^1,2^ or ustekinumab.^3^ Therefore, over time, a significant percentage of patients require a change in treatment. Unfortunately, there is little evidence to guide the choice of therapy, and clinicians are often left to decide based on their personal experience and by extracting information from some direct and indirect comparative efficacy studies with their limitations. This inefficient approach delays the control of inflammation and increases therapy risks and costs. Predicting early response is therefore crucial.

Predictive biomarkers are still in their infancy, and, until now, none has been implemented in clinical practice. Candidate biomarkers have experienced 2 main limitations, either not being drug-specific or lacking objective validation^4,5^: in the first case not guiding the choice of medication and in the second being difficult to generalize. As the target of IBD treatment evolves from clinical to endoscopic, and even further to histologic or transmural healing, ideal biomarkers should predict endoscopic remission.^6^ One emerging approach lies in identifying membrane-bound antibody labeling through probe-based confocal laser endomicroscopy (pCLE).^7,8^ pCLE involves passing a probe down the accessory channel of an endoscope, utilizing laser light striking a fluorescent contrast agent, injected intravenously before imaging, to provide histology-level imaging.^9,10^ A study by Atreya et al^7^ demonstrated how the visualization of membrane-bound TNF through fluorescent antibody labeling and in vivo CLE correlated with response to anti-TNF treatment. In another pilot study, CLE detection of fluorescent anti-α4β7 integrin antibodies predicted response to vedolizumab in 5 patients with a previous failure to anti-TNF,^8^ though these results are yet to be reproduced or validated.

Besides molecular labeling, pCLE provides the unique opportunity to evaluate the dynamic changes of the microscopic architecture in vivo.^11^ However, the interpretation of CLE imaging is challenging and thus far has been, with few exceptions, a prerogative of experienced endoscopists. Computer-aided quantitative analysis of image patterns has the potential to standardize CLE imaging and detect features of clinical relevance.^12,13^

Transcriptomics is another promising approach for the prediction of treatment response. Differences in gene expression analysis have been investigated to identify markers of specific disease phenotypes, including response to therapy.^14^ Transcriptomics can shed light on molecular pathway differences between remitters and nonremitters and suggest new possible biomarkers or targets for therapy. Similarly to the previous approaches, reproducibility and validation of discriminative molecular markers have been challenging partly due to disease heterogeneity.

This prospective study aimed to explore, in a multifaceted approach, potential predictive biomarkers of response to anti-TNF therapy and vedolizumab in ulcerative colitis (UC) and Crohn’s disease (CD), using pCLE, fluorescein isothiocyanate labeling, immunohistochemistry, and gene expression analysis.

## Methods

The study was approved by the Office for Research Ethics Committee Northern Ireland (Ref 17/NI/0148).

### Study setting and patients

We performed a prospective observational study at the University of Birmingham (United Kingdom). Patients with IBD (UC and CD) who were due to start a biological therapy as part of standard care were recruited. The inclusion criteria were patients ≥18 and ≤70 years of age with a known diagnosis of IBD and with evidence of ongoing inflammatory activity requiring biological therapy (infliximab/adalimumab or vedolizumab). Exclusion criteria were inability to provide consent, isolated small intestinal disease only, allergy to nuts or shellfish, serious comorbidities, toxic megacolon, renal failure, pregnancy or breastfeeding, severe or uncontrolled asthma, coagulopathy, previous allergic reaction to fluorescein, and concomitant use of beta-blockers.

### Study design

We recruited 29 IBD patients (14 with UC, 15 with CD) undergoing colonoscopy or flexible sigmoidoscopy prior to initiation of biological therapy. At the baseline procedure, demographic, medication, and clinical indices of disease activity data were collected before administration of the biologic agent. Twelve weeks after the initiation of anti-TNF therapy and 14 to 16 weeks following the start of vedolizumab, a follow-up colonoscopy or sigmoidoscopy was performed to evaluate endoscopic response. Endoscopic remission was defined as Mayo endoscopic score ≤1, Ulcerative Colitis Endoscopic Index of Severity (UCEIS) ≤1, and Paddington International virtual ChromoendoScopy ScOre (PICaSSO) ≤3 in UC and a reduction of ≥50% of the Simple Endoscopic Score for Crohn’s Disease (SES-CD) in CD. Partial response was defined as Mayo 2, UCEIS 2 to 4, PICaSSO 4 to 8, and 50% < SES-CD < 75% in CD and nonresponse as Mayo 3, UCEIS ≥7, and PICaSSO ≥8 in UC and SES-CD >75% in CD.

### Study objectives

We sought to identify endomicroscopy features predictive of biological therapy response; evaluate the correlation between mucosal expression of drug targets (TNF and α4β7 molecules) with response to the respective agents (anti-TNF therapy and vedolizumab) and develop predictive biomarkers for therapeutic response; and investigate differences in tissue ultrastructure, cellular immunophenotype, and gene expression between pre- and posttreatment.

### Endoscopic procedures

All endoscopic procedures, including pCLE, were performed by a single experienced endoscopist (M.I.), and the endoscopic findings were assessed by 3 additional gastroenterologists (O.M.N., R.C., S.C.L.S.). All procedures were performed using a 7010 Pentax Medical (Montvale, NJ, USA) processor, with high-definition white-light endoscopy and virtual chromoendoscopy (iScan 1, 2 and optical enhancement mode 1). Inflammatory activity was graded according to the Mayo endoscopic score,^15^ UCEIS,^16^ and the newly published PICaSSO score^17,18^ in UC and SES-CD^19^ in CD. Following identification of the most severely inflamed segment, intravenous fluorescein (2.5-5.0 mL) was injected to perform pCLE (Cellvizio; Mauna Kea Technologies, Paris, France) to further assess inflammatory activity. After the accurate and multimodal endoscopic assessment, targeted biopsies were taken in the most inflamed area. The histological activity was graded using the Robarts Histological Index (RHI) and^20,21^ Nancy Histological Index^22^ for UC and the modified Riley score and RHI for CD.^23^ Histological remission was defined as Nancy Histological Index ≤1,^22^ and RHI ≤3 without neutrophils in the epithelium and lamina propria.^20,21^ Additional biopsies for ex vivo imaging, immunohistochemistry, and gene expression analysis were taken in the same area assessed by pCLE.

### Ex vivo labeling

Drug formulations (infliximab and vedolizumab) were dialyzed overnight, and 1 mg of purified protein was labeled with fluorescein using the Fluorescein-EX Protein Labeling Kit F10240 (Thermo Fisher Scientific/Invitrogen, San Diego, CA, USA) according to the manufacturer’s instructions. Fractions containing labeled protein were pooled and adjusted to 1 mg/mL for ex vivo biopsy labeling for pCLE analysis of infliximab and vedolizumab binding.

Biopsies for ex vivo labeling were collected in phosphate-buffered saline and kept at 4°C for transport to the adjacent laboratory. After phosphate-buffered saline washing and incubating with the mucolytic N-acetyl glucosamine for 20 minutes at room temperature, biopsies were washed and incubated according to preoptimized conditions with fluorescein-conjugated drug (0.1 mg/mL) and with fluorescein IgG1 (isotype control) to exclude unspecific binding. For infliximab labeling, incubation was for 30 minutes at room temperature, while vedolizumab was labeled overnight at 4°C. Following labeling, biopsies were washed and imaged with ex vivo pCLE, whereby the confocal probe makes contact with the biopsy and images are obtained.

### Immunohistochemistry

Tissues were deparaffinized and rehydrated to water, and after a low-temperature retrieval technique, ALTER,^24^ they were immunostained on a Dako Autostainer (Dako, Stockport, UK). Briefly, staining comprised a 10-minute endogenous peroxidase block (Dako) followed by a 10-minute protein block in 2% casein (Vector Labs, Birmingham, UK). Sections were incubated in optimally diluted antibodies for 1 hour; mouse anti-TNFα, 1/200 (sc52746, Santa Cruz Biotechnology, CA, USA) and rabbit anti-integrin b7, 1/400 (HPA042277; Sigma-Aldrich, Gillingham, UK). Antibody detection was performed with Vector Excel mouse and rabbit kits, respectively and visualized in NovaRED chromagen (Vector Labs) for 5 minutes. All buffer washes performed were with EnVision FLEX wash buffer (Dako). Sections were then counterstained with Meyers hematoxylin, dehydrated through to xylene, and mounted with a glass coverslip in distyrene plasticizer xylene. Expression was scored as the following: 0 = none, 1 = low (<30%), 2 = medium (30%-60%), and 3 = high (>60%).

## Image analysis and quantification

### Video recording and image collections

All in vivo and ex vivo procedures were recorded, and still, mosaic images were reconstructed using CellvizioViewer (Mauna Kea Technologies).^25^ From the mosaic images, the following features were measured: pericrypt fluorescence, crypt diameter, intercrypt distance (ICD), wall thickness (WT) and fluorescein leakage through the colonic mucosa (FLCM) in the in vivo images and fluorescence intensity in the ex vivo images.

### Computer-aided pCLE image analysis

The post-CLE analysis was performed by 2 researchers (E.G., A.B.) blinded to clinical and endoscopic findings, using an in-home annotation software designed by MATLAB (R2021b, The Mathworks Inc, MA, USA) (The MathWorks, Inc., Natick, MA, USA) for manually outlining the visible crypts and the vessels^26^on mosaic images. Ten mosaic reconstructions per patient were analyzed. After the manual annotation, morphologic parameters were automatically evaluated, including vessel tortuosity, crypt distribution along the mucosal surface, crypt area, eccentricity, diameter, ICD, WT, and FLCM.^12,27^

### Computer-aided analysis of ex vivo labeling

Before quantifying the antigen binding, preprocessing was performed to remove obvious biases and artifacts derived from the optical acquisition and specimen preparation. Images from the same specimen were stacked, and all pixels with a standard intensity deviation smaller than a threshold $\vartheta_{\sigma }=0.01$ were discarded, as the slight variation across images indicates an acquisition artifact. On the remaining pixels of all images from the same specimen, the mean intensity value $\mu_{f\;\;luo}$ and SD of the intensity value $\sigma_{f\;\;luo}$were estimated, allowing the computation of a conservative threshold (fewer pixels selected) $\vartheta_{con}=\mu_{f\;\;luo}+2\sigma_{f\;\;luo}$, and a relaxed threshold$\vartheta_{rel}=\mu_{f\;\;luo}$. For each image, the pixels with intensity greater than $\vartheta_{con}$(and$\vartheta_{rel}$) were selected, and their area and average intensity were evaluated. Additionally, the average intensity of all pixels in the image was computed, as it might be correlated with the amount of fluorophores displayed.

## Gene expression analysis

### RNA extraction

Biopsies were transferred immediately to RNA later and stored below 4°C prior to on-column RNA extraction and purification using RNeasy columns (Qiagen, Hilden, Germany). Uniquely indexed complementary DNA libraries were prepared using QIAseq UPX 3ʹ Transcriptome reagents libraries (Qiagen) were quantified and quality-controlled using the QIAseq Library Quant Assay Kit and tapestation analysis (Qiagen). Sequencing was performed on MiSeq and NextSeq Illumina (San Diego, CA, USA) platforms. Libraries were de-multiplexed, genomically aligned, quantified, and normalized using the CLC Genomics Workbench (Qiagen). Gene expression in pretreatment samples of responders vs nonresponders was then compared.

### Differential gene expression and multivariate analysis

Genes with the TPM (transcripts per million) normalized values^28^ were log-transformed with a pseudo-count of 1 added. The limma package^29^ was employed for the differential expression analysis between conditions.^30^ The library size was estimated using a reduced maximum likelihood estimator with 500 iterations. The initial fitting was performed using a reduced maximum likelihood estimator (500 iterations), and the initial model fitting was performed using a robust M-estimation and moderated test statistics (empirical Bayes). An false discovery rate–corrected *P* value <.05 was considered and used for further downstream multivariate analysis. Partial least squares discriminant analysis modeling was performed on filtered genes, and a variable importance in projection (VIP) score was estimated to reduce the number of target genes, a VIP score of more than 1 was used.^31,32^

### Gene set enrichment analysis.

To better understand the pathways involved and their biological significance, an enrichment analysis was performed using the DAVID Gene Functional Classification Tool.^33^ This incorporates over 40 annotation categories, including Gene Ontology terms, protein–protein interactions, functional protein domains, disease associations, biological pathways, general sequence features, homologies, and tissue expressions to enrich the genes and cluster them based on the degrees of their co-association genes.^29,34^

### Gene external validation cohort

Publicly available gene expression data from the Gene Expression Omnibus database with accession number GSE 16879 were extracted. These data were used as a validation cohort. This cohort was based on 24 UC patients and 37 CD patients in whom mucosal RNA expression profiling was done before and after the first infliximab treatment.

### Statistical analysis

Descriptive statistics were reported as proportions for categorical data or mean ± SD for continuous variables. Pre- and posttreatment data from in vivo and ex vivo analysis were tested in the entire IBD cohort and the UC and CD subgroups.

Univariate logistic regression was used to define the optimal cutoffs for all measurements at baseline to identify responder patients. Given the small dataset available, leave-one-out cross-validation was performed, obtaining the predicted classification probability of all left-out samples. The performance of different parameters in predicting response was compared using area under the receiver-operating characteristic curve (AUROC), accuracy, positive predictive value (PPV), and negative predictive value (NPV).

## Results

### Patient characteristics

In total, 29 patients (15 with CD, 14 with UC) were enrolled in the study. The average age was 40 ± 12 years, 15 were men, and the mean disease duration was 12.2 years. Among UC patients, 6 had pancolitis, 7 had left-sided colitis, and 1 had proctitis; CD patients had colonic involvement in 8 cases and ileocolonic in the remaining 7, and 2 had previously undergone ileo-cecal resection. Overall, 21 (72%) of 29 were on steroids, 20 (69%) of 29 were on immunomodulators, 15 (52%) of 29 were on aminosalicylates, and only 4 (14%) were on a biologic. All had endoscopically active disease. Details of the patient characteristics and biologics used are presented in Tables 1 and 2.

**Table 1.** Patient demographics (N = 29)

Age , y	40.8 ± 12
Male	15 (51.7)
UC	14 (48.2)
CD	15 (51.8)
Extension of disease	
**UC**	
Proctitis	1 (7.1)
Left colitis	7 (50)
Pancolitis	6 (42.8)
**CD**	
Colonic	8 (53.3)
Ileocolonic	7 (46.7)
Baseline therapy	
Steroids	21 (72.4)
Aminosalicylates	15 (51.7)
Immunomodulators	20 (68.9)
Biologics	4 (13.8)
Optima biologics cohort	
Infliximab	7 (24.1)
Vedolizumab	5 (17.2)
Adalimumab	17 (58.6)
Outcome	
Responder	14 (48.3)
Partial responder	6 (20.7)
Nonresponder	9 (31)

Values are mean ± SD or n (%).

Abbreviations: CD, Crohn’s disease; UC, ulcerative colitis.

**Table 2.** Disease characteristics

Endoscopic Activity	Pretreatment	Posttreatment
Mayo endoscopic score		
Mayo 0	0	3 (21.4)
Mayo 1	2 (14.3)	5 (35.7)
Mayo 2	8 (57.1)	3 (21.4)
Mayo 3	4 (28.6)	3 (21.4)
UCEIS		
Remission (≤1)	0	5 (35.7)
Mild (2-4)	2 (14.3)	5 (35.7)
Moderate (5-7)	9 (64.3)	4 (28.6)
Severe (>7)	3 (21.4)	0
PICaSSO score		
Remission (≤3)	1 (7.1)	5 (35.7)
Active (≥3)	13 (92.9)	9 (64.3)
SES-CD (n = 13)		
Remission (0-2)	0	6 (46.2)
Mild (3-6)	1 (7.7)	2 (15.4)
Moderate (7-15)	11 (84.6)	5 (38.5)
Severe (>15)	1 (7.7)	0
Rutgeerts score (n = 2)		
Remission (i0-i1)	0	1 (50)
Moderate (i2)	0	0
Severe (i3-i4)	2 (100)	1 (50)

Values are n (%).

Abbreviations: PICaSSO, Paddington International virtual ChromoendoScopy ScOre; SES, Simple Endoscopic Score for Crohn’s Disease; UCEIS, Ulcerative Colitis Endoscopic Index of Severity.

## Computer-aided image analysis

### In vivo pCLE analysis

We analyzed 93 videos from 29 IBD patients before and after biological treatment, comprising 228 (range, 109-230) and 293 (range, 123-311) crypts, respectively. Vessel tortuosity was the only parameter across all patient data that was significantly altered (reduced) after treatment (*P* < .05). In UC, treatment significantly reduced FLCM (*P* < .05), whereas in CD patients it reduced crypts area, eccentricity, and ICD (*P* < .05) (Table 3). From the leave-one-out cross-validated logistic regression, crypt eccentricity was the most accurate discriminant between responders and nonresponders in the whole cohort, with an AUROC of 0.81, accuracy of 80%, PPV of 80%, and NPV of 80%, regardless of the biologic agent used. When considering UC alone, vessel tortuosity (AUROC, 0.93; accuracy 85%; PPV 89%; and NPV 75%), crypts area (AUROC, 1.0; accuracy 90%; PPV 100%; and NPV 90%), and eccentricity (AUROC, 0.88; accuracy 90%; PPV 86%; and NPV 100%) were the best discriminants of response. On the contrary, maximal pericryptic FLCM (AUROC, 0.79; accuracy 80%; PPV 75%; and NPV 83%), mean ICD (AUROC, 0.88; accuracy, 75%; PPV 50%; NPV 83%), and mean wall thickness (AUROC, 0.75%; accuracy 63%; PPV 33%; and NPV 80%) were the most predictive variables for CD. Unfortunately, a subgroup analysis with a different type of biologic in the UC and CD subgroups was not possible, given that only 1 CD patient was treated with vedolizumab.

**Table 3.** Summary of pCLE in vivo and ex vivo findings in responder vs nonresponder patients

		Pre–Post Variation (%)			Responder vs Nonresponder Identification (AUROC)		
		IBD	UC	CD	IBD	UC	CD
In vivo pCLE	Mean vessel tortuosity	-65^a^	-46	-78	0.52	0.93^a^	0.44
	Maximal crypts area	6	-16	29	0.65	1.00^a^	0.13
	Mean crypts eccentricity	4	-6	15^a^	0.81^a^	0.88^a^	0.54
	Maximal crypts diameter	1	-8	11	0.64	0.79^a^	0.50
	Mean ICD	8	-3	19^a^	0.66	0.33	0.88^a^
	Mean WT	6	-12	28	0.34	0.00	0.75^a^
	Maximal FLCM Pericryptic	66^a^	86^a^	-10	0.18	0.38	0.79^a^
	Mean FLCM elsewhere	-1	-15	10	0.54	0.68	0.68
Ex vivo pCLE	Mean high hyperfluorescent area	-18	10	-45	0.63	0.83^a^	0.58
	Mean high hyperfluorescent intensity	2	8^a,b^	-4	0.41	0.50	0.53
	Mean low hyperfluorescent area	-3	6	-11	0.46	0.80^a^	0.70^a^
	Mean low hyperfluorescent intensity	2	11	-6	0.53	0.20	0.65
	Maximal low hyperfluorescent intensity	2	14^a^	-8	0.49	0.60	0.70^a^
	Maximal low hyperfluorescent area	0	18	-15	0.48	0.53	0.03

Abbreviations: AUROC, area under the receiver-operating characteristic curve; CD, Crohn’s disease; FLCM, fluorescein leakage of colonic mucosa; IBD, inflammatory bowel disease; ICD, intercrypt distance; pCLE, probe confocal laser endomicroscopy; UC, ulcerative colitis; WT, wall thickness.

^a^ *P* < .05.

^b^ *P* = .06.

### Ex vivo pCLE molecular imaging with fluorescent biological agents before and after biological treatment

The binding of fluorescent labeled biological agents before and after treatment was analyzed ex vivo on mucosal biopsy specimens from UC and CD patients. An increased binding (area of the tissue) to the biological agent pretreatment was associated with a higher likelihood of response to the treatment. Interestingly, the magnitude of this prediction of response was greater in UC (AUROC, 83%; accuracy 77%; PPV 89%; NPV 50%) compared with CD (AUROC, 58%; accuracy 64%; PPV 40%; NPV 78%) (Table 3).

**Figure 1.**
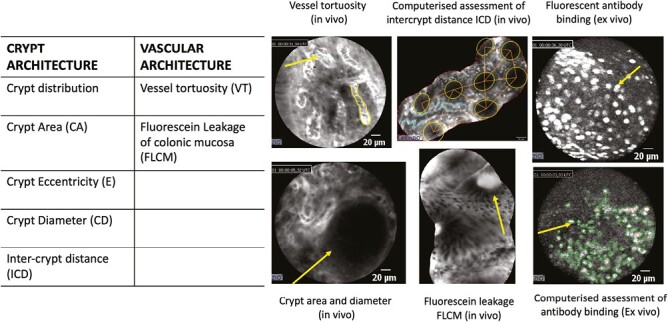
Summary of probe confocal laser endomicroscopy findings in vivo and ex vivo.

Across the overall population, there was no significant difference in the fluorescent intensity before and after treatment. However, UC patients had higher basal fluorescent intensity signals with a reduction, though not statistically significant, after treatment of 8% (*P* = .06) at the high threshold (high specificity for labeling) (Table 3). Among responders, UC patients had a significant reduction in fluorescent intensity of 14% (*P* < .05), whereas CD patients had no significant change. pCLE findings are summarized in Figure 1.

### Immunohistochemistry

**Figure 2.**
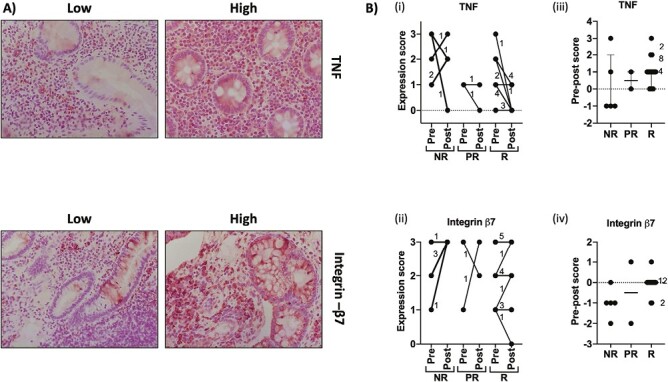
Immunohistochemistry for tumor necrosis factor (TNF) and integrin-β7 was performed on biopsies sampled before and after biologic treatment. Expression was scored on a scale of 0 to 3 in which 0 = none, 1 = low (<30%), 2 = medium (30%-60%), and 3 = high (>60%). **A,** Representative images of tissues with high and low expression. **B,** Summary of scores for tissues from patients classified as nonresponder (NR), partial responder (PR), and responder (R). i and ii indicate actual scores. iii and iv show reduction in score from pre- to posttreatment (pre score minus post score). Numbers indicate the number of patients represented where data points overlap.

Baseline expression of TNF protein did not significantly differ between responders and nonresponders. However, a decreased scoring of protein expression of TNF in responders compared with nonresponders was found, although this trend did not reach statistical significance. The number of patients treated with vedolizumab was limited in this study, and baseline expression did not relate to responder status (Figure 2).

### RNA sequencing

#### Differential gene expression analysis

**Figure 3.**
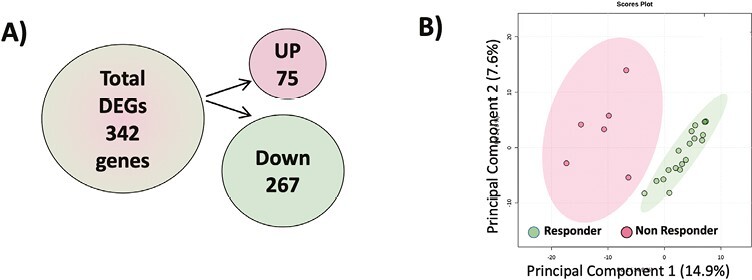
A, A diagrammatic representation of the direction of regulation of the differentially expressed genes (DEGs) when partial responders are considered as responders. B, A principal component analysis score plot performed on the 342 DEGs demonstrating clustering of the responders vs nonresponders. Dots represent patients and are colored according to the subject cohort. Ellipses represent 95% confidence.

To investigate potential gene networks that might underpin a patient’s response to anti-TNF therapy, we considered genes differentially expressed in the colons of responders vs nonresponders prior to therapy. Initially, partial responders were combined with responders and considered as overall responders. A total of 342 differently expressed genes (DEGs) with an adjusted *P* value <.05 and greater than 2-fold change were identified, of which 75 were upregulated and 267 downregulated (Figure 3A and Supplementary Table 1). A principal component analysis (PCA) showed a spatial separation of the responder vs nonresponder patients (Figure 3B). A partial least squares discriminant analysis further identified 143 genes VIP >1 (Supplementary Table 2). We also considered the potential of partial responders being classified as nonresponders. Although the PCA analysis resulted in a good separation of responders vs nonresponders, only 99 DEGs, 41 upregulated and 58 downregulated, were identified (Supplementary Figure 1 and Supplementary Table 1), and only 38 genes had VIP > 1 (Supplementary Table 2), suggesting that partial responders were more similar to responders. Hence, it was appropriate to consider them within the responder group.

#### Pathway enrichment analysis of DEGs

To further investigate the dysregulation of pathways within the colons of patients who fail to respond to anti-TNF treatment, a pathway enrichment analysis was performed on the 342 DEGs in responders vs nonresponders using the DAVID Functional Annotation Clustering tool (Supplementary Table 3). Pathways related to inflammation, focal adhesions and cell migration, extracellular matrix activity, guanyl-nucleotide exchange factor activity, and carbohydrate metabolism were enriched. Table 4 and Supplementary Figure 2 summarize the DEGs clustered within these pathways. In total, 76 DEGs fell within these pathways, including 15 upregulated and 61 downregulated targets. Overall, there was an apparent downregulation of inflammation-related genes in responders compared with nonresponders, including CXCL6 and CCL4L2, which are involved in leukocyte chemotaxis and HLA-DQB2, which is required for antigen presentation and T cell activation. Key regulators of inflammatory factors were also downregulated in responders, such as IRF7, which promotes the release of virus-induced type 1 interferons, and MAPKAPK2, which drives the release of inflammatory cytokines, including TNF after it is activated in response to stress, bacterial substrates, and inflammatory mediators. More vigorous activity of the TNF pathway in nonresponders pretreatment was also suggested by TNFAIP3 5-fold upregulation (adjusted *P* = .0047) combined with the higher expression of TNF shown by immunohistochemistry (Figure 2Bi and 2Biii). Further analysis of TNF in posttreatment biopsies suggested that response to treatment included downregulation of TNF, while for nonresponders TNF was increased posttreatment (Figure 2Bii). Likewise, in nonresponders, ITG-B7, the gene encoding integrin b7, increased posttreatment, while in responders it remained stable (Figure 2Biv). By RNA sequencing, responders also showed reduced extracellular matrix activity, evidenced by lower levels of ADMATS4, CRIP2, CTGF, and EMILIN1, while cell–cell adhesion was increased. Last, changes in metabolism were also observed, with reduced carbohydrate metabolism in responders.

**Table 4.** Pathway enriched differentially expressed genes in responders versus nonresponders to anti-TNFα therapy obtained using the DAVID Functional Annotation Clustering tool

		Focal Adhesion/Cytoskeleton and Migration	Extracellular Matrix	Cell Adhesion	Small GTPase Activity	Immune Response	MAP Kinase Signaling	Insulin Signaling	Carbohydrate Metabolism	Sterol Metabolism
VIP >1	Up	ACTN, ILK, RAC1		CXADR, EIF2A, PUF60, RPL14, YWHAB		AP2B1, ERAP1, YES1	STK4			PMVK
	Down	ARHGAP35, ARPC3, CIB1, LAMA4, PPP1R12B	ADAMTS4, CRIP2, CTGF, EMILIN1		DOCK7, DOCK8	AP2S1, CXCL6, CYR61, HLA-DQB2, JAK3, PXN, TGFBR1	GADD45B, MAPKAPK2		RPE, SLC3A1	FASN, MBTPS1
VIP <1	Up									EBP, HACD3
	Down	BRAF, FURIN, LAMB1, LIMK2, SLC9A1		BZW2, NEO1, PCDH1, SND1	ABR, ARFRP1, DIS3, MFHAS1, RABIF, RAPGEF1	BTN3A2, C1QA, C5AR1, CCL4L2, CHID1, CRK, GREM1, IRF7, KIF2A, SEC24A, SOCS4, SOCS6, TCF12, TNS3		PRKAG1	AKR1B1, GALC, GK5, HK1, MATN2	

Enriched genes are grouped according to their area of function and separated according to VIP score.

TNFα, tumor necrosis factor α; VIP, variable importance in projection.

#### Selection of markers predictive of anti-TNF response

**Figure 4.**
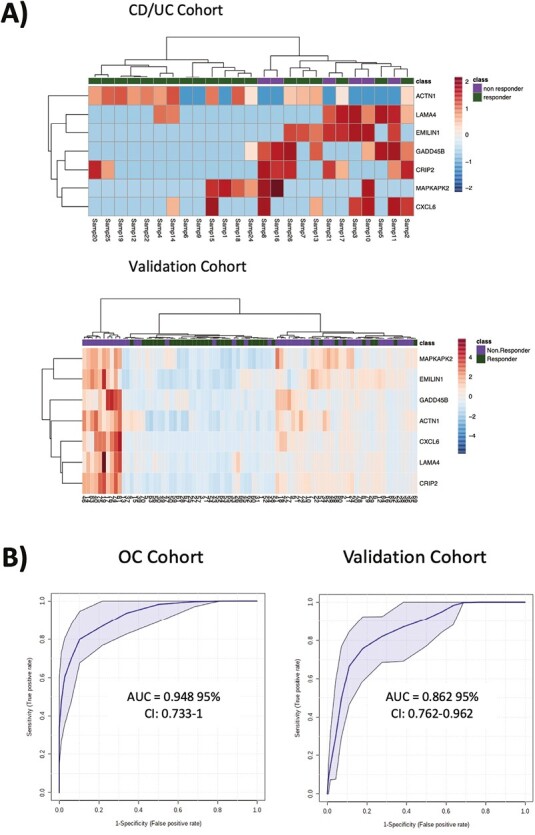
A selection of response predictive markers for anti-tumor necrosis factor (TNF) therapy. An area under the curve (AUC) analysis was performed on the enriched genes with variable importance in projection >1 that were also measured in the validation study. A, Heat map summaries of the 7 selected targets in the Crohn’s disease (CD)/ulcerative colitis (UC) and validation cohorts. B, Sensitivity vs specificity receiver-operating characteristic curves for combined use of CRIP2, CXCL6, EMILIN1, GADD45B, LAMA4, and MAPKAPK2 as predictors of TNF response in the optima cohort and validation cohorts. CI, confidence interval.

All 37 enriched genes with VIP >1 had an AUROC score of ≥0.7 (Supplementary Table 4). Therefore, to investigate these genes that might form the reliable predictors of anti-TNF response in IBD, we assessed their expression profile reported in a previously published total RNA expression dataset (GSE16879) that includes mucosal samples from 43 patients with IBD (UC and colonic CD) before treatment with infliximab. Responders in this cohort were classified according to their response to infliximab at 4 to 6 weeks. A total of 31 of our 37 enriched genes were also examined in this study, of which 7, including ACTN1, CRIP2, CXCL6, EMILIN1, GADD45B, LAMA4, and MAPKAPK2, had an AUCROC score of ≥0.7. As depicted in the heat maps in Figure 4A and the scatter box plots in Supplementary Figure 3, these selected targets, except ACTN1, exhibited common regulation patterns across the 2 cohorts. Partial least squares modeling using combined genes, provided area under the curve scores of 0.948 (95% confidence interval, 0.733-1) and 0.862 (95% confidence interval, 0.762-0.962) for the prediction of response in our optimal cohort and the validation cohort, respectively (Figure 4B), thus supporting their suitability as biomarkers of response to anti-TNF therapy. A quantitative polymerase chain reaction analysis of the expression of these 7 genes experimentally validated these findings, increasing their potential for being predictors of anti-TNF response in IBD.

**Figure 5.**
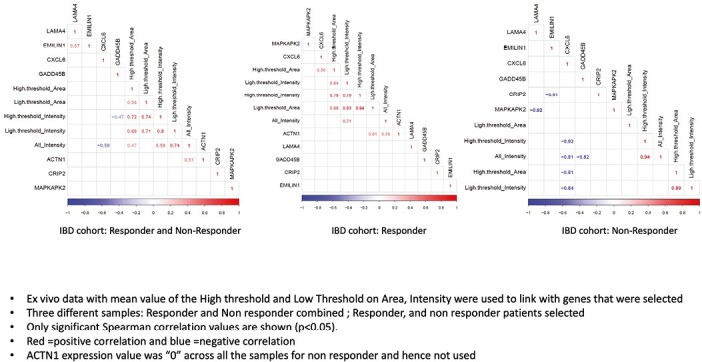
Correlation between ex vivo probe confocal laser endomicroscopy findings and genes. Ex vivo data with mean value of the high threshold and low threshold on area intensity were used to link with selected genes. Three different samples, responder and nonresponder combined, responder, and nonresponder, were selected. Only significant Spearman correlation values are shown (*P* < .05). Red = positive correlation; blue = negative correlation. The ACTN1 expression value was 0 across all the samples for nonresponder and hence was not used. IBD, inflammatory bowel disease.

Noteworthy, when correlating ex vivo imaging and gene expression, we observed a positive correlation (0.56—moderate but significant) between CXCL6 and the binding area intensity in responders and a strong negative correlation (-0.93) between CXCL6 and binding area intensity in nonresponders (Figure 5). The opposite correlation of the same gene from critical areas in patients with different responses supports a link between gene expression and ex vivo findings and the potential use of pCLE molecular imaging to probe cellular events such as leukocyte trafficking and activity.

## Discussion

In this study, we showed for the first time in vivo morphological and functional changes of crypt and microvasculature architecture after biological therapy by using a computer-aided pCLE image analysis. Upon treatment, we observed a significant reduction in FLCM in UC patients and a more regular arrangement with small round crypts in CD. Fluorescein leakage has been proposed as a pCLE marker for increased mucosal permeability. Among the pCLE findings, restoration of functional mucosal integrity measured as a reduction of FLCM could discriminate responders to biological treatment, irrespective of the drug’s mechanism of action. Importantly, we obtained these results through an automated analysis of pCLE images, thus overcoming interobserver variability, shortening the learning curve, and expediting assessment. Overall, by rendering the interpretation of pCLE and molecular endoscopy accessible, computer-aided quantitative analysis of image-based applications can streamline the adoption of pCLE and facilitate the identification and validation of mucosal markers for a therapeutic response. We have also recently shown that pCLE scoring reflected histological healing and used similar morphological and fluorescent leakage markers.^35^

A key finding of our study is the successful prediction of treatment response in UC through molecular labeling of drug targets. Previous works by Atreya et al^7^ and Rath et al^8^ showed how mucosal expression of TNF and α4β7 integrin correlated with response to adalimumab and vedolizumab, respectively. Building on this evidence, our study assessed the response to anti-TNF and anti-integrin treatment by fluorescent molecular imaging and, for the first time, showed a significant reduction in fluorescent intensity in UC treatment responders. This finding supports the use of molecular labeling for disease phenotypes screening and improvement of the upfront identification of patients benefiting from biological treatment. As such, these findings may represent a significant step toward personalized medicine. While immunohistochemistry for TNF and β7 integrins tended to support these findings, the results were not discriminatory or predictive of response. The weak correlation with the fluorescent molecular imaging findings might be explained by different antigen preservation between freshly collected and paraffin-embedded tissue. Previous studies reported a correlation between low tissue concentrations of anti-TNF and lack of response, suggesting that anti-TNF levels in nonresponders may be insufficient to neutralize the TNF^36,37^ Our results support the importance of the drug-to-target ratio.^37^ However, while it had been hypothesized that inflamed tissue leaked the drug reducing its concentration, our pathway enrichment analysis of DEGs showed a higher pretreatment activity of the TNF pathway in nonresponders. In other words, nonresponders expressed a more inflammatory burden regardless of the levels of anti-TNF mucosal binding. This suggests that TNF overexpression, rather than anti-TNF leaking, might be an essential cause of the low anti-TNF-to-TNF ratio in nonresponders. Thus, higher drug levels, at least of infliximab, may overcome such nonresponse, though the case for higher adalimumab levels remains conflicting in light of the recent SERENE trials (Study of a Novel Approach to Induction and Maintenance Dosing With Adalimumab in Patients With Moderate to Severe Ulcerative Colitis) showing no benefits of higher induction and maintenance doses.^38,39^

Furthermore, our work sheds light on the differences in gene expression between anti-TNF responders and nonresponders. The 7-gene panel we identified as the best predictor resulted in an excellent diagnostic performance, supporting possible clinical use, pending further validation across the larger patient population. These genes are involved in pathways such as inflammation, chemotaxis, transforming growth factor β signaling, and extracellular matrix, biologically consistent with a differential expression in IBD. Moreover, 4 of these genes, namely CXCL6,^14,40,41^ EMILIN1-β1,^42^ GADD45B,^43^ and MAPKAPK2,^44,45^ had been previously associated with IBD. As gene profiling revolutionized oncological treatment choices, it may in the future have a similar impact on complex multifactorial diseases, such as IBD.^46^

The gene panel identified has biological plausibility to inflammation and response to drugs in IBD, especially UC. Chemokines play a crucial role in the recruitment and function of neutrophils, hence the relevance of CXCL6 in neutrophil trafficking and activation-mediated disease such as UC, interacting with receptors CXCR1 and CXCR2.^40^ Laminin family members, such as LAMA4, interact with members of the integrin family, promoting cell adhesion and trafficking field.^47^ EMILIN1-β1 and integrin interactions in the extracellular vascular matrix are essential in mediating colitis in animal models.^42^ Both constitutive androstane receptor and pregnane X receptor ameliorate experimental colitis through GADD45B. Constitutive androstane receptor and pregnane X receptor cooperatively ameliorate dextran sulfate sodium–induced colitis,^48,49^ as shown by us and others. Finally, MAPKAPK2 is involved in gut inflammatory pathways and regulation of TNF gene expression, and its inhibition results in an improvement in animal models of colitis.^50^

Our study has certain limitations. First, the small sample size limits the generalizability of the results. We recruited patients requiring biologic therapy, mostly refractory to immunosuppressant and steroids. Most of the participants received anti-TNF therapies, the most common first line of biologic treatment. The choice to assess vedolizumab efficacy after 14 to 16 weeks instead of 12 as for anti-TNF was based on data from clinical trials including the VARSITY (Vedolizumab versus Adalimumab for Moderate-to-Severe Ulcerative Colitis) and VERDICT (In actiVE ulcerative colitis, a RanDomIzed Controlled Trial for determination of the optimal treatment target) studies.^51-53^ As classes of targeted therapies expand, future studies will need to include them, and account for bio-naive and bio-exposed patients to better reflect clinical practice.

Second, the whole labeling process, although feasible, is complicated in the absence of standardized guidance on fluorescein labeling, storage, and application to the tissue. Quantifying immunofluorescence in ex vivo pCLE is also challenging. The technique we used was developed as a proof of concept for this study in the absence of validated methods, and the weak correlation (disparity) with immunohistochemistry could be related to unspecific binding. However, the direct labeling of the biological agent providing less background fluorescence and inclusion of isotype control confirm the specificity of the fluorescent signals obtained. Third, although the gene panel we identified had a high prognostic value, the reproducibility of such gene expression results is often limited. In the past, the overlap between differentially expressed genes in similar studies has been modest,^14^ hindering their practical application.

In addition, probe confocal laser microscopy undoubtedly requires training, and its costs have limited its implementation in clinical practice. However, it provides both dynamic ultrastructural and vascular details in real life and molecular details after labeling, which may provide predictive information after targeted therapies. Computer-aided analysis enabled by artificial intelligence has the potential to help the operator interpret images. Thus, it is expected to provide decision support to make clinical applications easier to implement. It will also harmonize and standardize the read-outs in future and reduce subjectiveness.

Further, new imaging with fluorescent molecular probes integrated with the endoscopes is in early phase development. It might be the future on the horizon that could support endoscopists with real-time visualization of targeted and tailoring therapy.

## Conclusions

Quantitative computer-aided image analysis of pCLE in vivo and assessing the binding of fluorescent-labeled biologics ex vivo accurately predicted response to biologic treatment in IBD, particularly in UC. At baseline, anti-TNF responders and nonresponders had a significantly different expression of genes mainly involved in the inflammatory cascade. However, further prospective studies are required to confirm these preliminary results.

## Supplementary Material

izac233_suppl_Supplementary_Figure_S1

izac233_suppl_Supplementary_Figure_S2

izac233_suppl_Supplementary_Figure_S3

izac233_suppl_Supplementary_Table_S1

izac233_suppl_Supplementary_Table_S2

izac233_suppl_Supplementary_Table_S3

izac233_suppl_Supplementary_Table_S4

## References

[1] Ben-Horin S , Kopylov U, Chowers Y. Optimizing anti-TNF treatments in inflammatory bowel disease. Autoimmun Rev. 2014;13(1):24-30. 23792214 10.1016/j.autrev.2013.06.002

[2] Amiot A , Grimaud J-C, Peyrin-Biroulet L, et al.; Observatory on Efficacy and of Vedolizumab in Patients With Inflammatory Bowel Disease Study Group. Effectiveness and safety of vedolizumab induction therapy for patients with inflammatory bowel disease. Clin Gastroenterol Hepatol. 2016;14(11):1593-1601.e2. 26917043 10.1016/j.cgh.2016.02.016

[3] Wils P , Bouhnik Y, Michetti P, et al; Groupe d'Etude Thérapeutique des Affections Inflammatoires du Tube Digestif. Subcutaneous ustekinumab provides clinical benefit for two-thirds of patients with crohn’s disease refractory to anti-tumor necrosis factor agents. Clin Gastroenterol Hepatol. 2016;14(2):242-50.e1-2. 26432476 10.1016/j.cgh.2015.09.018

[4] Stevens TW , Matheeuwsen M, Lönnkvist MH, et al. Systematic review: predictive biomarkers of therapeutic response in inflammatory bowel disease-personalised medicine in its infancy. Aliment Pharmacol Ther. 2018;48(11-12):1213-1231. 30378142 10.1111/apt.15033

[5] Boyapati RK , Kalla R, Satsangi J, Ho G-T. Biomarkers in search of precision medicine in IBD. Am J Gastroenterol. 2016;111(12):1682-1690. 27670602 10.1038/ajg.2016.441

[6] Turner D , Ricciuto A, Lewis A, et al.; International Organization for the Study of IBD. STRIDE-II: an update on the Selecting Therapeutic Targets in Inflammatory Bowel Disease (STRIDE) Initiative of the International Organization for the Study of IBD (IOIBD): determining therapeutic goals for treat-to-target strategies in IBD. Gastroenterology 2021;160(5):1570-1583. 33359090 10.1053/j.gastro.2020.12.031

[7] Atreya R , Neumann H, Neufert C, et al. In vivo imaging using fluorescent antibodies to tumor necrosis factor predicts therapeutic response in Crohn’s disease. Nat Med. 2014;20(3):313-318. 24562382 10.1038/nm.3462 PMC4479137

[8] Rath T , Bojarski C, Neurath MF, Atreya R. Molecular imaging of mucosal α4β7 integrin expression with the fluorescent anti-adhesion antibody vedolizumab in Crohn’s disease. Gastrointest Endosc. 2017;86(2):406-408. 28137597 10.1016/j.gie.2017.01.012

[9] Rasmussen DN , Karstensen JG, Riis LB, Brynskov J, Vilmann P. Confocal laser endomicroscopy in inflammatory bowel disease--a systematic review. J Crohns Colitis 2015;9(12):1152-1159. 26209861 10.1093/ecco-jcc/jjv131

[10] Neumann H , Vieth M, Atreya R, et al. Assessment of Crohn’s disease activity by confocal laser endomicroscopy. Inflamm Bowel Dis. 2012;18(12):2261-2269. 22344873 10.1002/ibd.22907

[11] Chapman CG , Konda VJA. Confocal laser endomicroscopy in inflammatory bowel disease: achieving new depths in mucosal healing. Gastrointest Endosc. 2016;83(4):792-794. 26975284 10.1016/j.gie.2015.11.002

[12] Quénéhervé L , David G, Bourreille A, et al. Quantitative assessment of mucosal architecture using computer-based analysis of confocal laser endomicroscopy in inflammatory bowel diseases. Gastrointest Endosc. 2019;89(3):626-636. 30120955 10.1016/j.gie.2018.08.006

[13] Schneider Y , Buchner AM. Computer-aided confocal laser endomicroscopy in inflammatory bowel disease: probing deeper into what it means. Gastrointest Endosc. 2019;89(3):637-638. 30784501 10.1016/j.gie.2018.11.005

[14] Arijs I , Li K, Toedter G, et al. Mucosal gene signatures to predict response to infliximab in patients with ulcerative colitis. Gut 2009;58(12):1612-1619. 19700435 10.1136/gut.2009.178665

[15] Schroeder KW , Tremaine WJ, Ilstrup DM. Coated oral 5-aminosalicylic acid therapy for mildly to moderately active ulcerative colitis. A randomized study. N Engl J Med. 1987;317(26):1625-1629. 3317057 10.1056/NEJM198712243172603

[16] Travis SPL , Schnell D, Krzeski P, et al. Developing an instrument to assess the endoscopic severity of ulcerative colitis: the Ulcerative Colitis Endoscopic Index of Severity (UCEIS). Gut 2012;61(4):535-542. 21997563 10.1136/gutjnl-2011-300486 PMC3292713

[17] Iacucci M , Smith SCL, Bazarova A, et al. An international multicenter real-life prospective study of electronic chromoendoscopy score PICaSSO in ulcerative colitis. Gastroenterology 2021;160(5):1558-1569.e8. 33347880 10.1053/j.gastro.2020.12.024

[18] Iacucci M , Daperno M, Lazarev M, et al. Development and reliability of the new endoscopic virtual chromoendoscopy score: the PICaSSO (Paddington International Virtual ChromoendoScopy ScOre) in ulcerative colitis. Gastrointest Endosc. 2017;86(6):1118-1127.e5. 28322774 10.1016/j.gie.2017.03.012

[19] Daperno M , D’Haens G, Van Assche G, et al. Development and validation of a new, simplified endoscopic activity score for Crohn’s disease: the SES-CD. Gastrointest Endosc. 2004;60(4):505-512. 15472670 10.1016/s0016-5107(04)01878-4

[20] Mosli MH , Feagan BG, Zou G, et al. Development and validation of a histological index for UC. Gut 2017;66(1):50-58. 26475633 10.1136/gutjnl-2015-310393

[21] Pai RK , Khanna R, D’Haens GR, et al. Definitions of response and remission for the Robarts Histopathology Index. Gut 2019;68(11):2101-2102. 30366909 10.1136/gutjnl-2018-317547

[22] Marchal-Bressenot A , Salleron J, Boulagnon-Rombi C, et al. Development and validation of the Nancy histological index for UC. Gut 2017;66(1):43-49. 26464414 10.1136/gutjnl-2015-310187

[23] Feagan BG , Greenberg GR, Wild G, et al. Treatment of ulcerative colitis with a humanized antibody to the alpha4beta7 integrin. N Engl J Med. 2005;352(24):2499-2507. 15958805 10.1056/NEJMoa042982

[24] Reynolds GM , Visentin B, Sabbadini R. Immunohistochemical detection of sphingosine-1-phosphate and sphingosine kinase-1 in human tissue samples and cell lines. Methods Mol Biol. 2018;1697:43-56. 28560513 10.1007/7651_2017_44

[25] Becker V , Vercauteren T, von Weyhern CH, et al. High-resolution miniprobe-based confocal microscopy in combination with video mosaicing (with video). Gastrointest Endosc. 2007;66(5):1001-1007. 17767932 10.1016/j.gie.2007.04.015

[26] Veronese E , Poletti E, Buda A, et al. Semiautomatic evaluation of crypt architecture and vessel morphology in confocal microendoscopy: application to ulcerative colitis. In: Roa Romero LM, ed. XIII Mediterranean Conference on Medical and Biological Engineering and Computing 2013. IFMBE Proceedings. Cham, Switzerland: Springer International; 2014:435-438.

[27] Buda A , Hatem G, Neumann H, et al. Confocal laser endomicroscopy for prediction of disease relapse in ulcerative colitis: a pilot study. J Crohn’s Colitis. 2014;8(4):304-311. 24094597 10.1016/j.crohns.2013.09.005

[28] Li B , Ruotti V, Stewart RM, Thomson JA, Dewey CN. RNA-Seq gene expression estimation with read mapping uncertainty. Bioinformatics 2010;26(4):493-500. 20022975 10.1093/bioinformatics/btp692 PMC2820677

[29] Huang DW , Sherman BT, Lempicki RA. Bioinformatics enrichment tools: paths toward the comprehensive functional analysis of large gene lists. Nucleic Acids Res. 2009;37(1):1-13. 19033363 10.1093/nar/gkn923 PMC2615629

[30] Ritchie ME , Phipson B, Wu D, et al. limma powers differential expression analyses for RNA-sequencing and microarray studies. Nucleic Acids Res. 2015;43(7):e47. 25605792 10.1093/nar/gkv007 PMC4402510

[31] Iacucci M , Jeffery L, Acharjee A, et al. Ultra-high magnification endocytoscopy and molecular markers for defining endoscopic and histologic remission in ulcerative colitis—an exploratory study to define deep remission. Inflamm Bowel Dis. 2021;27(11):1719-1730. 34019073 10.1093/ibd/izab059 PMC8528147

[32] Liu K , Acharjee A, Hinz C, et al. Consequences of lipid remodeling of adipocyte membranes being functionally distinct from lipid storage in obesity. J Proteome Res. 2020;19(10):3919-3935. 32646215 10.1021/acs.jproteome.9b00894

[33] Chen EY , Tan CM, Kou Y, et al. Enrichr: interactive and collaborative HTML5 gene list enrichment analysis tool. BMC Bioinformatics. 2013;14:128. 23586463 10.1186/1471-2105-14-128 PMC3637064

[34] Huang DW , Sherman BT, Lempicki RA. Systematic and integrative analysis of large gene lists using DAVID bioinformatics resources. Nat Protoc. 2009;4(1):44-57. 19131956 10.1038/nprot.2008.211

[35] Iacucci M , Cannatelli R, Gui X, et al. Assessment of endoscopic healing by using advanced technologies reflects histological healing in ulcerative colitis. J Crohns Colitis 2020;14(9):1282-1289. 32201877 10.1093/ecco-jcc/jjaa056

[36] Olsen T , Goll R, Cui G, et al. Tissue levels of tumor necrosis factor-alpha correlates with grade of inflammation in untreated ulcerative colitis. Scand J Gastroenterol. 2007;42(11):1312-1320. 17852866 10.1080/00365520701409035

[37] Yarur AJ , Jain A, Sussman DA, et al. The association of tissue anti-TNF drug levels with serological and endoscopic disease activity in inflammatory bowel disease: the ATLAS study. Gut 2016;65(2):249-255. 25670812 10.1136/gutjnl-2014-308099

[38] D’Haens GR , Sandborn WJ, Loftus EV, et al. Higher vs standard adalimumab induction dosing regimens and two maintenance strategies: randomized SERENE CD trial results. Gastroenterology 2022;162(7):1876-1890. 35122766 10.1053/j.gastro.2022.01.044

[39] Panés J , Colombel J-F, D’Haens GR, et al. Higher vs standard adalimumab induction and maintenance dosing regimens for treatment of ulcerative colitis: SERENE UC trial results. Gastroenterology 2022;162(7):1891-1910. 35227777 10.1053/j.gastro.2022.02.033

[40] Gijsbers K , Van Assche G, Joossens S, et al. CXCR1-binding chemokines in inflammatory bowel diseases: downregulated IL-8/CXCL8 production by leukocytes in Crohn’s disease and selective GCP-2/CXCL6 expression in inflamed intestinal tissue. Eur J Immunol. 2004;34(7):1992-2000. 15214047 10.1002/eji.200324807

[41] Shi L , Han X, Li J-X, et al. Identification of differentially expressed genes in ulcerative colitis and verification in a colitis mouse model by bioinformatics analyses. World J Gastroenterol. 2020;26(39):5983-5996. 33132649 10.3748/wjg.v26.i39.5983 PMC7584051

[42] Capuano A , Pivetta E, Sartori G, et al. Abrogation of EMILIN1-β1 integrin interaction promotes experimental colitis and colon carcinogenesis. Matrix Biol. 2019;83:97-115. 31479698 10.1016/j.matbio.2019.08.006

[43] Kumar A , Malhotra P, Coffing H, et al. Epigenetic modulation of intestinal Na+/H+ exchanger-3 expression. Am J Physiol Gastrointest Liver Physiol. 2018;314(3):G309-G318. 29167115 10.1152/ajpgi.00293.2017 PMC5899241

[44] Strasser SD , Ghazi PC, Starchenko A, et al. Substrate-based kinase activity inference identifies MK2 as driver of colitis. Integr Biol (Camb). 2019;11(7):301-314. 31617572 10.1093/intbio/zyz025 PMC7208439

[45] Wang Z , Liang XY, Chang X, et al. MMI-0100 ameliorates dextran sulfate sodium-induced colitis in mice through targeting MK2 pathway. Molecules. 2019;24(15):2832. 31382637 10.3390/molecules24152832 PMC6696270

[46] Bravo-Merodio L , Acharjee A, Russ D, et al. Translational biomarkers in the era of precision medicine. Adv Clin Chem. 2021;102:191-232. 34044910 10.1016/bs.acc.2020.08.002

[47] Katagiri F , Ishikawa M, Yamada Y, Hozumi K, Kikkawa Y, Nomizu M. Screening of integrin-binding peptides from the laminin α4 and α5 chain G domain peptide library. Arch Biochem Biophys. 2012;521(1-2):32-42. 22391228 10.1016/j.abb.2012.02.017

[48] Uehara D , Tojima H, Kakizaki S, et al. Constitutive androstane receptor and pregnane X receptor cooperatively ameliorate DSS-induced colitis. Dig Liver Dis. 2019;51(2):226-235. 30442521 10.1016/j.dld.2018.10.008

[49] Hudson GM , Flannigan KL, Erickson SL, et al. Constitutive androstane receptor regulates the intestinal mucosal response to injury. Br J Pharmacol. 2017;174(12):1857-1871. 28320072 10.1111/bph.13787 PMC5446585

[50] Thuraisingam T , Xu YZ, Moisan J, et al. Distinct role of MAPKAPK-2 in the regulation of TNF gene expression by Toll-like receptor 7 and 9 ligands. Mol Immunol. 2007;44(14):3482-3491. 17485113 10.1016/j.molimm.2007.03.019

[51] Sands BE , Peyrin-Biroulet L, Loftus EV, et al.; VARSITY Study Group. Vedolizumab versus adalimumab for moderate-to-severe ulcerative colitis. N Engl J Med. 2019;381(13):1215-1226. 31553834 10.1056/NEJMoa1905725

[52] Peyrin-Biroulet L , Loftus EV, Jr, Colombel JF, et al. Histologic outcomes with vedolizumab versus adalimumab in ulcerative colitis: results from an Efficacy and Safety Study of Vedolizumab Intravenous Compared to Adalimumab Subcutaneous in Participants With Ulcerative Colitis (VARSITY). Gastroenterology 2021;161(4):1156-1167.e3. 34144047 10.1053/j.gastro.2021.06.015

[53] Danese S , Sandborn WJ, Colombel J-F, et al. Endoscopic, radiologic, and histologic healing with vedolizumab in patients with active Crohn’s disease. Gastroenterology 2019;157(4):1007-1018.e7. 31279871 10.1053/j.gastro.2019.06.038

