# Supplementary material for: Computer-Aided Imaging Analysis of Probe-Based Confocal Laser Endomicroscopy With Molecular Labeling and Gene Expression Identifies Markers of Response to Biological Therapy in IBD Patients: The Endo-Omics Study
Source: Inflamm Bowel Dis. 2022 Nov 15;29(9):1409–20. doi: 10.1093/ibd/izac233 (PMC10472745; doi:10.1093/ibd/izac233)
Supplement: izac233_suppl_Supplementary_Figure_S1 [file izac233_suppl_supplementary_figure_s1.pdf]

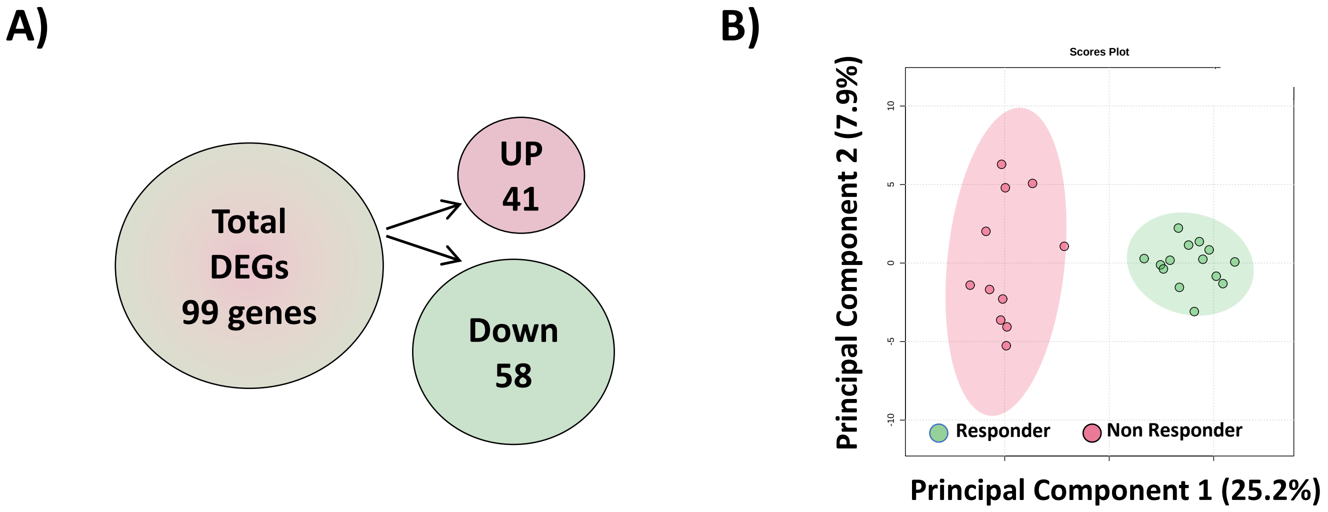

Supplementary figure 1. A) Diagrammatic representation of the direction of regulation of the differentially expressed genes (DEGs) when partial responders are considered as non-responders. **B)** Principal component analysis (PCA) score plot performed on the 99 DEGs. Dots represent patients and are coloured according to the subject cohort. Ellipses represent 95% confidence.
