# Supplementary material for: Computer-Aided Imaging Analysis of Probe-Based Confocal Laser Endomicroscopy With Molecular Labeling and Gene Expression Identifies Markers of Response to Biological Therapy in IBD Patients: The Endo-Omics Study
Source: Inflamm Bowel Dis. 2022 Nov 15;29(9):1409–20. doi: 10.1093/ibd/izac233 (PMC10472745; doi:10.1093/ibd/izac233)
Supplement: izac233_suppl_Supplementary_Figure_S3 [file izac233_suppl_supplementary_figure_s3.pdf]

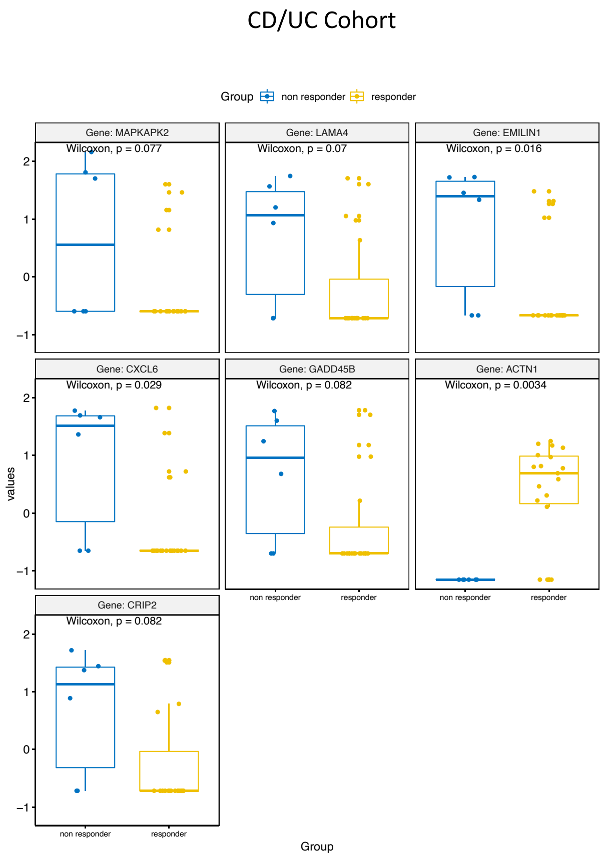

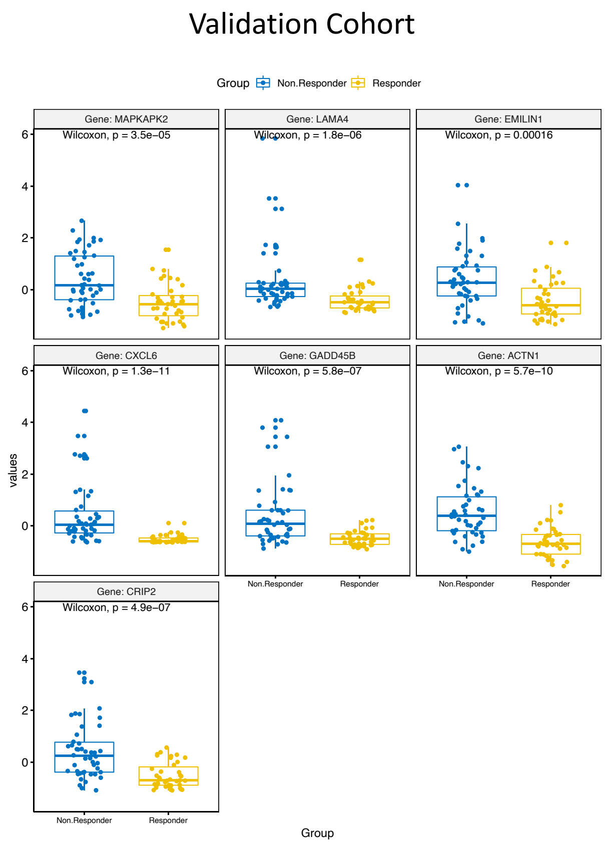

Supplementary figure 3: Box plot summaries for the 7 selected targets in the CD/UC and validation cohorts
