# Supplementary material for: Computer-Aided Imaging Analysis of Probe-Based Confocal Laser Endomicroscopy With Molecular Labeling and Gene Expression Identifies Markers of Response to Biological Therapy in IBD Patients: The Endo-Omics Study
Source: Inflamm Bowel Dis. 2022 Nov 15;29(9):1409–20. doi: 10.1093/ibd/izac233 (PMC10472745; doi:10.1093/ibd/izac233)
Supplement: izac233_suppl_Supplementary_Table_S1 [file izac233_suppl_supplementary_table_s1.docx]

**Supplementary Table 1**: Differentially expressed genes in anti-TNF𝛼-treated CD/UC patients compared as i) responders + partial responders vs non-responders and ii) responders vs partial responders + non-responders.

| Responder + Partial responder vs Non-responder | | | |  | Responder vs Partial responder + Non-responder | | | |
| --- | --- | --- | --- | --- | --- | --- | --- | --- |
| Down-regulated | | Upregulated | |  | Down-regulated | | Upregulated | |
| Gene | logFC | Gene | logFC |  | Gene | logFC | Gene | logFC |
| RBM6 | -13,0732 | EBP | 2,085134 |  | SMUG1 | -10,4866 | CDC42SE1 | 3,029745 |
| TAF4 | -13,0195 | NPM1 | 2,402716 |  | MTPN | -10,4468 | EDF1 | 3,158644 |
| CLIC4 | -12,4363 | SAP18 | 2,526212 |  | JAK3 | -10,2163 | TCF25 | 5,701817 |
| CXCL6 | -12,3564 | RAC1 | 2,737525 |  | N4BP2 | -10,209 | PFDN6 | 6,239657 |
| PAM | -12,3407 | YWHAB | 2,79225 |  | SH3RF1 | -10,1829 | RAB7A | 6,367578 |
| CRYL1 | -12,2837 | MPC2 | 2,905416 |  | ZNFX1 | -9,85367 | CEMIP2 | 6,372059 |
| EIF2AK3 | -12,2557 | CSDE1 | 3,0421 |  | BTF3L4 | -9,6876 | CAAP1 | 6,571806 |
| LOC101927741 | -12,0725 | CLASP1 | 3,053768 |  | FLCN | -9,66873 | CKS2 | 6,6509 |
| IP6K2 | -11,8435 | RPL14 | 3,292187 |  | FAM107B | -9,54553 | NIPSNAP1 | 6,662708 |
| MNT | -11,7474 | IRF2BP2 | 3,650922 |  | CD300A | -9,3604 | RBM7 | 6,70558 |
| HTT | -11,4699 | NAXE | 5,363809 |  | FAM49A | -9,34979 | FAXDC2 | 7,045654 |
| NHLRC3 | -11,2813 | SARS | 5,402214 |  | GADD45B | -9,28193 | EMP2 | 7,288512 |
| TTN | -11,2126 | EIF3K | 5,439897 |  | GMFB | -9,28057 | RNH1 | 7,427841 |
| PIM1 | -11,1694 | UGT2B17 | 5,532518 |  | CCM2 | -9,24372 | EVI5 | 7,432029 |
| EMILIN1 | -11,1664 | SMIM26 | 5,662552 |  | PXN | -9,23968 | CCDC59 | 7,710937 |
| BHLHE40 | -11,1187 | ACP1 | 5,78481 |  | CPD | -9,14471 | ERVK3-1 | 7,716778 |
| CARS2 | -11,1136 | ATP8B1 | 5,789185 |  | ZCCHC7 | -8,77012 | MCM4 | 7,770734 |
| ZBTB4 | -11,0921 | USP34 | 6,014735 |  | EMILIN1 | -8,5938 | SNAP47 | 7,814067 |
| RPE | -11,0766 | PHB | 6,04013 |  | ANPEP | -8,53988 | NXT1 | 7,857967 |
| PRIM2 | -10,9816 | RBMX | 6,090826 |  | RFLNB | -8,47873 | OXNAD1 | 7,878492 |
| MAFK | -10,9289 | AKR1C3 | 6,123923 |  | BASP1 | -8,44561 | SPINT1 | 8,062742 |
| METTL15 | -10,8289 | SNRPB2 | 6,247493 |  | ZCRB1 | -8,42429 | L1TD1 | 8,220814 |
| C5AR1 | -10,7632 | EIF4A3 | 6,377418 |  | LILRA5 | -8,40298 | ZFR | 8,319408 |
| VPS33A | -10,7622 | HACD3 | 6,386927 |  | SACM1L | -8,27503 | R3HDM2 | 8,466596 |
| ENTPD1 | -10,7477 | CSNK1A1 | 6,461843 |  | COL12A1 | -8,08125 | CXADR | 8,508797 |
| RBM28 | -10,721 | TXN2 | 6,572075 |  | FPR1 | -7,91458 | CEP78 | 8,669028 |
| FASN | -10,7072 | GSTO1 | 6,59463 |  | CTSK | -7,88725 | EFNA1 | 8,9296 |
| XPNPEP3 | -10,7043 | PUF60 | 6,614289 |  | JARID2 | -7,79557 | SP3 | 9,096879 |
| ADAMTS4 | -10,6164 | NFIB | 6,647032 |  | PLIN2 | -7,56059 | ZNF281 | 9,153631 |
| TNS3 | -10,6051 | PHB2 | 6,872635 |  | BMS1 | -7,15162 | GOLGA2 | 9,334773 |
| LOC100049716 | -10,5805 | VPS35 | 6,944806 |  | LOC105379382 | -7,14114 | TMEM245 | 9,56219 |
| DOCK8 | -10,5647 | DHRS3 | 6,963606 |  | TRIM74 | -7,13255 | RSF1 | 9,585825 |
| PXN | -10,5298 | CWC15 | 7,002962 |  | POLR3GL | -6,96506 | IFT172 | 9,603769 |
| LAMA4 | -10,5202 | PDZD8 | 7,029737 |  | STAU2 | -6,93917 | OGT | 9,78665 |
| DOCK7 | -10,5027 | GPX1 | 7,127531 |  | PHC2 | -6,89888 | RSBN1 | 9,964926 |
| TGFBR1 | -10,4976 | CDC37 | 7,178615 |  | CNN1 | -6,66931 | LXN | 10,04994 |
| BRWD3 | -10,4873 | EIF2A | 7,206667 |  | MRPL47 | -6,60337 | DAZAP1 | 10,18449 |
| STAU2 | -10,4784 | ILK | 7,218257 |  | RPSAP58 | -6,45785 | ARID4B | 10,45609 |
| PPP1R12B | -10,4137 | MRPS18B | 7,261517 |  | CXCL6 | -6,4316 | SLC22A18AS | 10,59355 |
| EIF5A2 | -10,4055 | ETNK1 | 7,376764 |  | MGP | -5,83251 | ACTN1 | 10,75403 |
| PCCB | -10,3563 | OPTN | 7,503921 |  | ZBTB4 | -5,70203 | PMVK | 11,14135 |
| APPBP2 | -10,3483 | NOP16 | 7,595966 |  | OAZ2 | -5,2585 |  |  |
| CUL4A | -10,3267 | SSFA2 | 7,604086 |  | NEK7 | -4,79718 |  |  |
| UBTF | -10,2756 | COX14 | 7,621321 |  | RAB8A | -3,56297 |  |  |
| ZNF395 | -10,2517 | HINT2 | 7,725468 |  | HIF1A-AS1 | -2,70812 |  |  |
| RPLP0P2 | -10,2063 | MBOAT1 | 7,847661 |  | ABCB10 | -2,48139 |  |  |
| SLC12A7 | -10,1966 | ERAP1 | 7,910297 |  | ZNF467 | -2,39091 |  |  |
| MARS2 | -10,1594 | MESD | 7,932805 |  | KRT6A | -2,35339 |  |  |
| ECPAS | -10,1417 | SLC44A4 | 8,02375 |  | ST3GAL6 | -2,33611 |  |  |
| GADD45B | -10,1013 | RNH1 | 8,069477 |  | POLG2 | -2,20673 |  |  |
| SND1 | -10,0501 | STK4 | 8,069559 |  | FCRL1 | -2,19531 |  |  |
| MFHAS1 | -10,0269 | PKIB | 8,10953 |  | SPINK1 | -2,19128 |  |  |
| ARHGAP35 | -9,97026 | YES1 | 8,11476 |  | CBL | -2,1697 |  |  |
| PRRC2B | -9,90855 | HNRNPA0 | 8,394505 |  | SNX16 | -2,14957 |  |  |
| TWIST1 | -9,88419 | SEC61A1 | 8,490613 |  | SYNJ2BP-COX16 | -2,11908 |  |  |
| MBTPS1 | -9,86752 | MRPL17 | 8,510804 |  | NHS | -2,07262 |  |  |
| CRIP2 | -9,84069 | SENP6 | 8,636392 |  | SNX33 | -2,06201 |  |  |
| MINK1 | -9,75789 | SMC2 | 8,643768 |  | LIPT2 | -2,05402 |  |  |
| DUSP10 | -9,64002 | EEA1 | 8,654451 |  |  |  |  |  |
| YARS2 | -9,60284 | CEMIP2 | 8,716761 |  |  |  |  |  |
| SHMT1 | -9,51937 | AP2B1 | 8,73069 |  |  |  |  |  |
| LSM8 | -9,48446 | EMP2 | 8,733603 |  |  |  |  |  |
| C11orf49 | -9,44142 | MAOA | 8,768402 |  |  |  |  |  |
| ANKLE2 | -9,37957 | SETD3 | 8,776195 |  |  |  |  |  |
| IKBIP | -9,2931 | PMVK | 8,820194 |  |  |  |  |  |
| CTGF | -9,19502 | ARID2 | 8,848285 |  |  |  |  |  |
| SIN3A | -9,19213 | FCF1 | 8,877633 |  |  |  |  |  |
| MARVELD3 | -9,17976 | EVI5 | 9,118651 |  |  |  |  |  |
| PDK4 | -9,17672 | JMJD1C | 9,384835 |  |  |  |  |  |
| CNN1 | -9,05318 | C20orf197 | 9,455688 |  |  |  |  |  |
| BZW2 | -9,04851 | POGZ | 9,783269 |  |  |  |  |  |
| MED1 | -9,00318 | BBX | 9,839867 |  |  |  |  |  |
| CPD | -8,99919 | TMC5 | 9,840155 |  |  |  |  |  |
| HLA-DQB2 | -8,88197 | CXADR | 9,961742 |  |  |  |  |  |
| TCF12 | -8,85992 | ACTN1 | 10,11683 |  |  |  |  |  |
| MCM3 | -8,84508 |  |  |  |  |  |  |  |
| NDE1 | -8,76734 |  |  |  |  |  |  |  |
| LRBA | -8,76208 |  |  |  |  |  |  |  |
| AKIRIN1 | -8,74414 |  |  |  |  |  |  |  |
| STIM2 | -8,71618 |  |  |  |  |  |  |  |
| RNF113A | -8,69629 |  |  |  |  |  |  |  |
| MCCC2 | -8,67457 |  |  |  |  |  |  |  |
| ARFRP1 | -8,65968 |  |  |  |  |  |  |  |
| TXLNA | -8,65116 |  |  |  |  |  |  |  |
| COQ4 | -8,6389 |  |  |  |  |  |  |  |
| RNPEPL1 | -8,59315 |  |  |  |  |  |  |  |
| DNTTIP1 | -8,57066 |  |  |  |  |  |  |  |
| LIMK2 | -8,56382 |  |  |  |  |  |  |  |
| ALG1 | -8,55571 |  |  |  |  |  |  |  |
| ESRRA | -8,53791 |  |  |  |  |  |  |  |
| CHRNE | -8,50435 |  |  |  |  |  |  |  |
| GREM1 | -8,475 |  |  |  |  |  |  |  |
| GMFB | -8,46195 |  |  |  |  |  |  |  |
| IRF7 | -8,45231 |  |  |  |  |  |  |  |
| SLC35B1 | -8,42673 |  |  |  |  |  |  |  |
| COBL | -8,39575 |  |  |  |  |  |  |  |
| RHBDD2 | -8,3897 |  |  |  |  |  |  |  |
| RPSAP58 | -8,38655 |  |  |  |  |  |  |  |
| SACM1L | -8,38375 |  |  |  |  |  |  |  |
| MFSD10 | -8,34789 |  |  |  |  |  |  |  |
| B3GALNT2 | -8,28675 |  |  |  |  |  |  |  |
| ERF | -8,28172 |  |  |  |  |  |  |  |
| CYR61 | -8,28116 |  |  |  |  |  |  |  |
| TRMT13 | -8,26563 |  |  |  |  |  |  |  |
| LEPROTL1 | -8,25957 |  |  |  |  |  |  |  |
| DUSP11 | -8,24066 |  |  |  |  |  |  |  |
| TMX4 | -8,21224 |  |  |  |  |  |  |  |
| TATDN3 | -8,21061 |  |  |  |  |  |  |  |
| RABIF | -8,18987 |  |  |  |  |  |  |  |
| SEC24A | -8,13047 |  |  |  |  |  |  |  |
| SCAF8 | -8,1265 |  |  |  |  |  |  |  |
| C21orf2 | -8,12041 |  |  |  |  |  |  |  |
| ATPAF1 | -8,08861 |  |  |  |  |  |  |  |
| ACSS2 | -8,0767 |  |  |  |  |  |  |  |
| CHTF8 | -8,06133 |  |  |  |  |  |  |  |
| TSKU | -8,0507 |  |  |  |  |  |  |  |
| SLC3A1 | -8,03313 |  |  |  |  |  |  |  |
| FAM193A | -7,9363 |  |  |  |  |  |  |  |
| MS4A8 | -7,93374 |  |  |  |  |  |  |  |
| NFAT5 | -7,93275 |  |  |  |  |  |  |  |
| RCE1 | -7,92256 |  |  |  |  |  |  |  |
| TNIP2 | -7,9197 |  |  |  |  |  |  |  |
| PTP4A3 | -7,87591 |  |  |  |  |  |  |  |
| TMEM184B | -7,79026 |  |  |  |  |  |  |  |
| TIMM10B | -7,76518 |  |  |  |  |  |  |  |
| PRPSAP1 | -7,75346 |  |  |  |  |  |  |  |
| TCTN2 | -7,73861 |  |  |  |  |  |  |  |
| KAT6B | -7,73303 |  |  |  |  |  |  |  |
| WDR48 | -7,62961 |  |  |  |  |  |  |  |
| LIMD2 | -7,62602 |  |  |  |  |  |  |  |
| STX16 | -7,61584 |  |  |  |  |  |  |  |
| E2F4 | -7,59673 |  |  |  |  |  |  |  |
| SPI1 | -7,59071 |  |  |  |  |  |  |  |
| PRPF38B | -7,58758 |  |  |  |  |  |  |  |
| SOCS6 | -7,51941 |  |  |  |  |  |  |  |
| PAPD4 | -7,48233 |  |  |  |  |  |  |  |
| ARFGAP1 | -7,41796 |  |  |  |  |  |  |  |
| SERTAD1 | -7,36026 |  |  |  |  |  |  |  |
| BCL11A | -7,30763 |  |  |  |  |  |  |  |
| COA5 | -7,24867 |  |  |  |  |  |  |  |
| MAT2A | -7,23995 |  |  |  |  |  |  |  |
| DBR1 | -7,13645 |  |  |  |  |  |  |  |
| COX7A1 | -7,11655 |  |  |  |  |  |  |  |
| C3orf70 | -7,06625 |  |  |  |  |  |  |  |
| SERTAD3 | -7,05898 |  |  |  |  |  |  |  |
| NUDT7 | -7,04019 |  |  |  |  |  |  |  |
| KIF2A | -7,01503 |  |  |  |  |  |  |  |
| AGFG1 | -6,98061 |  |  |  |  |  |  |  |
| PRADC1 | -6,96614 |  |  |  |  |  |  |  |
| DNAJC12 | -6,87877 |  |  |  |  |  |  |  |
| IMMT | -6,87804 |  |  |  |  |  |  |  |
| DNAJB4 | -6,76701 |  |  |  |  |  |  |  |
| ANKRD44 | -6,75425 |  |  |  |  |  |  |  |
| LACTB | -6,70713 |  |  |  |  |  |  |  |
| MTX2 | -6,69902 |  |  |  |  |  |  |  |
| MATN2 | -6,67741 |  |  |  |  |  |  |  |
| JAK3 | -6,66766 |  |  |  |  |  |  |  |
| AQP1 | -6,66116 |  |  |  |  |  |  |  |
| ELK3 | -6,63793 |  |  |  |  |  |  |  |
| SOCS4 | -6,5565 |  |  |  |  |  |  |  |
| STEAP1 | -6,53908 |  |  |  |  |  |  |  |
| HK1 | -6,50695 |  |  |  |  |  |  |  |
| MFSD5 | -6,40614 |  |  |  |  |  |  |  |
| MCPH1 | -6,40251 |  |  |  |  |  |  |  |
| GALC | -6,24158 |  |  |  |  |  |  |  |
| ST8SIA4 | -6,23429 |  |  |  |  |  |  |  |
| DHX9 | -6,21803 |  |  |  |  |  |  |  |
| F13A1 | -6,18075 |  |  |  |  |  |  |  |
| KIAA1468 | -6,11675 |  |  |  |  |  |  |  |
| C19orf25 | -6,05978 |  |  |  |  |  |  |  |
| C5orf15 | -6,01658 |  |  |  |  |  |  |  |
| ANKRD39 | -5,98726 |  |  |  |  |  |  |  |
| BNIP2 | -5,94133 |  |  |  |  |  |  |  |
| SLF2 | -5,9252 |  |  |  |  |  |  |  |
| RNF41 | -5,9067 |  |  |  |  |  |  |  |
| PRKAG1 | -5,56009 |  |  |  |  |  |  |  |
| TIMM44 | -5,49322 |  |  |  |  |  |  |  |
| AKR1B1 | -5,47502 |  |  |  |  |  |  |  |
| SNRPA1 | -5,41729 |  |  |  |  |  |  |  |
| FLAD1 | -5,37303 |  |  |  |  |  |  |  |
| MRPL28 | -5,36635 |  |  |  |  |  |  |  |
| CD83 | -5,24231 |  |  |  |  |  |  |  |
| CRK | -5,1918 |  |  |  |  |  |  |  |
| DIS3 | -5,12395 |  |  |  |  |  |  |  |
| PCDH1 | -4,92967 |  |  |  |  |  |  |  |
| MAP4K4 | -4,8648 |  |  |  |  |  |  |  |
| BRAF | -4,85737 |  |  |  |  |  |  |  |
| CCL4L2 | -4,82237 |  |  |  |  |  |  |  |
| CNTRL | -4,70492 |  |  |  |  |  |  |  |
| PIGH | -4,70208 |  |  |  |  |  |  |  |
| MED28 | -4,57092 |  |  |  |  |  |  |  |
| NFYB | -4,52434 |  |  |  |  |  |  |  |
| CUTA | -4,3907 |  |  |  |  |  |  |  |
| SNRPF | -4,37407 |  |  |  |  |  |  |  |
| RSPO3 | -4,36686 |  |  |  |  |  |  |  |
| SMIM31 | -4,35931 |  |  |  |  |  |  |  |
| NEO1 | -4,3551 |  |  |  |  |  |  |  |
| CIB1 | -4,31578 |  |  |  |  |  |  |  |
| ARPC3 | -4,26675 |  |  |  |  |  |  |  |
| ELP5 | -4,26159 |  |  |  |  |  |  |  |
| MRPL22 | -4,14577 |  |  |  |  |  |  |  |
| IFI35 | -4,09649 |  |  |  |  |  |  |  |
| EVADR | -4,04228 |  |  |  |  |  |  |  |
| UMPS | -4,00232 |  |  |  |  |  |  |  |
| NEK7 | -3,80857 |  |  |  |  |  |  |  |
| CD37 | -3,80081 |  |  |  |  |  |  |  |
| PPCDC | -3,74896 |  |  |  |  |  |  |  |
| SLC9A1 | -3,66461 |  |  |  |  |  |  |  |
| ABR | -3,63035 |  |  |  |  |  |  |  |
| CTSB | -3,60995 |  |  |  |  |  |  |  |
| TNPO3 | -3,55566 |  |  |  |  |  |  |  |
| NSD1 | -3,54786 |  |  |  |  |  |  |  |
| UGT1A6 | -3,54499 |  |  |  |  |  |  |  |
| DMAC1 | -3,47757 |  |  |  |  |  |  |  |
| DDHD1 | -3,47426 |  |  |  |  |  |  |  |
| CAMKMT | -3,38284 |  |  |  |  |  |  |  |
| ZDHHC9 | -3,36659 |  |  |  |  |  |  |  |
| MAPKAPK2 | -3,36585 |  |  |  |  |  |  |  |
| ATXN2 | -3,35023 |  |  |  |  |  |  |  |
| GATAD1 | -3,26775 |  |  |  |  |  |  |  |
| UBE4A | -3,23917 |  |  |  |  |  |  |  |
| GPR107 | -3,23893 |  |  |  |  |  |  |  |
| AP2S1 | -3,23563 |  |  |  |  |  |  |  |
| PRC1 | -3,18048 |  |  |  |  |  |  |  |
| ECHDC2 | -3,16589 |  |  |  |  |  |  |  |
| MYNN | -3,15542 |  |  |  |  |  |  |  |
| LOC105370481 | -3,13011 |  |  |  |  |  |  |  |
| RAPGEF1 | -3,04937 |  |  |  |  |  |  |  |
| AFAP1L2 | -3,00983 |  |  |  |  |  |  |  |
| ELP6 | -2,99022 |  |  |  |  |  |  |  |
| CBFA2T2 | -2,98036 |  |  |  |  |  |  |  |
| UBR4 | -2,97105 |  |  |  |  |  |  |  |
| ZDHHC16 | -2,96592 |  |  |  |  |  |  |  |
| LAMB1 | -2,86129 |  |  |  |  |  |  |  |
| ABHD12 | -2,85539 |  |  |  |  |  |  |  |
| TERF1 | -2,85088 |  |  |  |  |  |  |  |
| CAMSAP2 | -2,83192 |  |  |  |  |  |  |  |
| BTN3A2 | -2,75848 |  |  |  |  |  |  |  |
| C1QA | -2,6919 |  |  |  |  |  |  |  |
| TRAPPC6B | -2,67475 |  |  |  |  |  |  |  |
| RBM47 | -2,66388 |  |  |  |  |  |  |  |
| CHID1 | -2,65173 |  |  |  |  |  |  |  |
| AK4 | -2,61571 |  |  |  |  |  |  |  |
| FBXL15 | -2,61437 |  |  |  |  |  |  |  |
| WBP1 | -2,56931 |  |  |  |  |  |  |  |
| ALAD | -2,52707 |  |  |  |  |  |  |  |
| FURIN | -2,50484 |  |  |  |  |  |  |  |
| NFATC2IP | -2,49187 |  |  |  |  |  |  |  |
| RNASEH2B | -2,48721 |  |  |  |  |  |  |  |
| TMEM126A | -2,4849 |  |  |  |  |  |  |  |
| GABPB1 | -2,47615 |  |  |  |  |  |  |  |
| SNX14 | -2,47175 |  |  |  |  |  |  |  |
| CBWD5 | -2,46671 |  |  |  |  |  |  |  |
| CCNYL1 | -2,45034 |  |  |  |  |  |  |  |
| FAM13A | -2,4063 |  |  |  |  |  |  |  |
| SLC16A1 | -2,34812 |  |  |  |  |  |  |  |
| TNFAIP3 | -2,33639 |  |  |  |  |  |  |  |
| SECISBP2 | -2,32095 |  |  |  |  |  |  |  |
| CETN3 | -2,31456 |  |  |  |  |  |  |  |
| SF3B3 | -2,27793 |  |  |  |  |  |  |  |
| EXOSC6 | -2,25098 |  |  |  |  |  |  |  |
| RNF216 | -2,22926 |  |  |  |  |  |  |  |
| CCZ1P-OR7E38P | -2,20422 |  |  |  |  |  |  |  |
| SLC22A23 | -2,19493 |  |  |  |  |  |  |  |
| CDC20 | -2,18248 |  |  |  |  |  |  |  |
| WWOX | -2,17923 |  |  |  |  |  |  |  |
| GK5 | -2,16208 |  |  |  |  |  |  |  |
|  |  |  |  |  |  |  |  |  |
