# Supplementary material for: Computer-Aided Imaging Analysis of Probe-Based Confocal Laser Endomicroscopy With Molecular Labeling and Gene Expression Identifies Markers of Response to Biological Therapy in IBD Patients: The Endo-Omics Study
Source: Inflamm Bowel Dis. 2022 Nov 15;29(9):1409–20. doi: 10.1093/ibd/izac233 (PMC10472745; doi:10.1093/ibd/izac233)
Supplement: izac233_suppl_Supplementary_Table_S2 [file izac233_suppl_supplementary_table_s2.docx]

**Supplementary Table 2:**

Variable Importance in Projection (VIP) scores for differentially expressed genes in anti-TNF𝛼-treated CD/UC patients compared as i) responders + partial responders vs non-responders and ii) responders vs partial responders + non-responders.

| **Responder +  Partial responder vs  Non-responder** | | **Responder vs  Partial responder +  Non-responder** | |
| --- | --- | --- | --- |
| **Gene** | **VIP score** | **Gene** | **VIP score** |
| RAC1 | 1,9204 | FAM107B | 1,5311 |
| ACTN1 | 1,8316 | N4BP2 | 1,4457 |
| PKIB | 1,7362 | CD300A | 1,443 |
| ARPC3 | 1,7187 | PMVK | 1,4128 |
| PMVK | 1,6857 | EFNA1 | 1,4083 |
| MRPL17 | 1,6625 | ACTN1 | 1,389 |
| RNH1 | 1,6406 | LXN | 1,3871 |
| TAF4 | 1,6316 | CEP78 | 1,3302 |
| ZNF395 | 1,6028 | OGT | 1,3287 |
| AKR1C3 | 1,5843 | CEMIP2 | 1,3243 |
| CTSB | 1,5729 | ANPEP | 1,2966 |
| METTL15 | 1,5697 | L1TD1 | 1,2938 |
| TMC5 | 1,5563 | SACM1L | 1,2894 |
| STAU2 | 1,5514 | ZCRB1 | 1,2856 |
| ZBTB4 | 1,5494 | IFT172 | 1,2829 |
| JMJD1C | 1,5301 | ZNFX1 | 1,2599 |
| COQ4 | 1,5172 | SMUG1 | 1,2327 |
| HINT2 | 1,4921 | JAK3 | 1,1834 |
| TRMT13 | 1,4896 | CCDC59 | 1,1625 |
| ST8SIA4 | 1,4826 | GADD45B | 1,1612 |
| RSPO3 | 1,4537 | FAXDC2 | 1,1529 |
| NHLRC3 | 1,4478 | ZNF281 | 1,1485 |
| CUTA | 1,4453 | OXNAD1 | 1,1395 |
| RBM6 | 1,4434 | RAB8A | 1,1325 |
| CRYL1 | 1,4419 | ARID4B | 1,131 |
| DMAC1 | 1,4399 | RNH1 | 1,1285 |
| SENP6 | 1,4365 | SLC22A18AS | 1,1206 |
| DOCK8 | 1,4246 | SH3RF1 | 1,0727 |
| EMP2 | 1,4245 | MTPN | 1,0717 |
| CDC37 | 1,4216 | PFDN6 | 1,071 |
| PAM | 1,4159 | ERVK3-1 | 1,0695 |
| MPC2 | 1,4081 | GOLGA2 | 1,0671 |
| MRPL22 | 1,4071 | SPINT1 | 1,0639 |
| TGFBR1 | 1,4021 | STAU2 | 1,0502 |
| NPM1 | 1,3979 | CAAP1 | 1,0406 |
| CYR61 | 1,3954 | SNAP47 | 1,0216 |
| PUF60 | 1,3901 | SP3 | 1,0181 |
| CXCL6 | 1,3778 | PLIN2 | 1 |
| GSTO1 | 1,3778 | CPD | 0,99625 |
| RBM47 | 1,3768 | RSBN1 | 0,98075 |
| DNTTIP1 | 1,3641 | CXCL6 | 0,97611 |
| TTN | 1,364 | CDC42SE1 | 0,97492 |
| NOP16 | 1,3588 | R3HDM2 | 0,97263 |
| YWHAB | 1,3555 | RBM7 | 0,95146 |
| FCF1 | 1,3539 | PXN | 0,95021 |
| SLC44A4 | 1,3474 | CKS2 | 0,94985 |
| CEMIP2 | 1,3456 | EMILIN1 | 0,94788 |
| DUSP11 | 1,3451 | POLR3GL | 0,94506 |
| BHLHE40 | 1,3263 | FAM49A | 0,93878 |
| LOC100049716 | 1,3245 | DAZAP1 | 0,92936 |
| MAFK | 1,3217 | GMFB | 0,92149 |
| SETD3 | 1,3164 | EVI5 | 0,92148 |
| NFIB | 1,3111 | ABCB10 | 0,91998 |
| CNN1 | 1,3099 | EMP2 | 0,91928 |
| CXADR | 1,3058 | ZFR | 0,91497 |
| EMILIN1 | 1,3024 | NXT1 | 0,9115 |
| ARHGAP35 | 1,2991 | LOC105379382 | 0,90918 |
| EVI5 | 1,2902 | ZNF467 | 0,90487 |
| BBX | 1,28 | SNX16 | 0,89926 |
| FASN | 1,2749 | FLCN | 0,89873 |
| CWC15 | 1,2683 | NEK7 | 0,89596 |
| ETNK1 | 1,2657 | FPR1 | 0,88709 |
| YES1 | 1,2655 | TCF25 | 0,88571 |
| SCAF8 | 1,2625 | CXADR | 0,87733 |
| RBM28 | 1,2582 | RSF1 | 0,87181 |
| DNAJC12 | 1,2466 | CCM2 | 0,86986 |
| EIF2A | 1,2464 | RAB7A | 0,8682 |
| HNRNPA0 | 1,2453 | ZCCHC7 | 0,86689 |
| GPX1 | 1,2445 | MCM4 | 0,85728 |
| STK4 | 1,244 | PHC2 | 0,85364 |
| EIF5A2 | 1,2369 | BTF3L4 | 0,84375 |
| PIM1 | 1,2337 | SPINK1 | 0,82398 |
| RPL14 | 1,2298 | ZBTB4 | 0,82392 |
| ARID2 | 1,2251 | EDF1 | 0,82331 |
| DOCK7 | 1,2176 | RFLNB | 0,81434 |
| VPS33A | 1,2041 | CNN1 | 0,80559 |
| ACP1 | 1,203 | NIPSNAP1 | 0,79227 |
| SEC61A1 | 1,2005 | TMEM245 | 0,78398 |
| PCCB | 1,1972 | MGP | 0,78059 |
| UGT1A6 | 1,1882 | BASP1 | 0,75685 |
| SACM1L | 1,1839 | BMS1 | 0,75486 |
| SMIM26 | 1,1825 | ST3GAL6 | 0,73689 |
| XPNPEP3 | 1,1753 | LIPT2 | 0,73641 |
| BCL11A | 1,1716 | FCRL1 | 0,73605 |
| SMC2 | 1,1706 | CBL | 0,73601 |
| CIB1 | 1,1662 | KRT6A | 0,73595 |
| RCE1 | 1,1658 | SNX33 | 0,73508 |
| RPE | 1,16 | HIF1A-AS1 | 0,73365 |
| FAM193A | 1,1599 | NHS | 0,73044 |
| ATP8B1 | 1,1588 | SYNJ2BP-COX16 | 0,72893 |
| SIN3A | 1,1542 | COL12A1 | 0,7098 |
| CTGF | 1,1541 | POLG2 | 0,70867 |
| DHRS3 | 1,1524 | CTSK | 0,68728 |
| AP2B1 | 1,1488 | TRIM74 | 0,68282 |
| PHB | 1,1461 | RPSAP58 | 0,67841 |
| AP2S1 | 1,1457 | JARID2 | 0,61056 |
| MESD | 1,1451 | LILRA5 | 0,58751 |
| ILK | 1,1421 | OAZ2 | 0,57694 |
| PHB2 | 1,1407 | MRPL47 | 0,55751 |
| PDK4 | 1,1374 |  |  |
| IP6K2 | 1,1338 |  |  |
| CLIC4 | 1,1312 |  |  |
| MAOA | 1,128 |  |  |
| SLC35B1 | 1,1257 |  |  |
| PAPD4 | 1,1242 |  |  |
| SARS | 1,1228 |  |  |
| SNRPF | 1,1227 |  |  |
| ADAMTS4 | 1,1156 |  |  |
| CSNK1A1 | 1,1106 |  |  |
| AQP1 | 1,1097 |  |  |
| PPP1R12B | 1,1017 |  |  |
| TWIST1 | 1,1001 |  |  |
| RNF113A | 1,0996 |  |  |
| MBTPS1 | 1,0894 |  |  |
| JAK3 | 1,089 |  |  |
| NAXE | 1,0864 |  |  |
| ERAP1 | 1,0847 |  |  |
| SLC3A1 | 1,0842 |  |  |
| SSFA2 | 1,0837 |  |  |
| TMX4 | 1,0835 |  |  |
| TXN2 | 1,0819 |  |  |
| EEA1 | 1,0804 |  |  |
| TIMM10B | 1,0777 |  |  |
| MARS2 | 1,0767 |  |  |
| PXN | 1,076 |  |  |
| LAMA4 | 1,0668 |  |  |
| LRBA | 1,0637 |  |  |
| MNT | 1,0635 |  |  |
| ECPAS | 1,0628 |  |  |
| CRIP2 | 1,0579 |  |  |
| GADD45B | 1,0351 |  |  |
| MAPKAPK2 | 1,0322 |  |  |
| SAP18 | 1,0312 |  |  |
| HTT | 1,0265 |  |  |
| SNRPA1 | 1,0262 |  |  |
| PTP4A3 | 1,0262 |  |  |
| VPS35 | 1,0229 |  |  |
| PRADC1 | 1,0225 |  |  |
| ESRRA | 1,013 |  |  |
| LOC101927741 | 1,0127 |  |  |
| PRPSAP1 | 1,008 |  |  |
| TERF1 | 1,0067 |  |  |
| HLA-DQB2 | 1,0013 |  |  |
| TNS3 | 0,9964 |  |  |
| UGT2B17 | 0,99344 |  |  |
| TMEM126A | 0,99234 |  |  |
| MBOAT1 | 0,98203 |  |  |
| TXLNA | 0,97863 |  |  |
| COX14 | 0,9728 |  |  |
| POGZ | 0,97267 |  |  |
| DBR1 | 0,97181 |  |  |
| CNTRL | 0,97032 |  |  |
| MTX2 | 0,96895 |  |  |
| CLASP1 | 0,96222 |  |  |
| MFHAS1 | 0,96208 |  |  |
| RBMX | 0,95547 |  |  |
| GMFB | 0,95249 |  |  |
| BRAF | 0,94484 |  |  |
| STX16 | 0,94425 |  |  |
| STIM2 | 0,94415 |  |  |
| ELK3 | 0,94235 |  |  |
| ATPAF1 | 0,94198 |  |  |
| UBTF | 0,93806 |  |  |
| LEPROTL1 | 0,93667 |  |  |
| CARS2 | 0,9325 |  |  |
| RNPEPL1 | 0,928 |  |  |
| IRF2BP2 | 0,92561 |  |  |
| CD83 | 0,92424 |  |  |
| CUL4A | 0,92179 |  |  |
| MINK1 | 0,91855 |  |  |
| PRRC2B | 0,91696 |  |  |
| ERF | 0,91362 |  |  |
| MRPL28 | 0,91034 |  |  |
| DUSP10 | 0,9084 |  |  |
| PRKAG1 | 0,9063 |  |  |
| LOC105370481 | 0,90232 |  |  |
| YARS2 | 0,89808 |  |  |
| C11orf49 | 0,89425 |  |  |
| ACSS2 | 0,89191 |  |  |
| MRPS18B | 0,89085 |  |  |
| NFYB | 0,88902 |  |  |
| ABR | 0,88728 |  |  |
| SERTAD1 | 0,88598 |  |  |
| ENTPD1 | 0,8792 |  |  |
| CPD | 0,87493 |  |  |
| SF3B3 | 0,86991 |  |  |
| GPR107 | 0,86223 |  |  |
| SNRPB2 | 0,85998 |  |  |
| TCTN2 | 0,85949 |  |  |
| MFSD10 | 0,85813 |  |  |
| ECHDC2 | 0,85585 |  |  |
| MCCC2 | 0,85272 |  |  |
| RNASEH2B | 0,85142 |  |  |
| STEAP1 | 0,84107 |  |  |
| KAT6B | 0,83991 |  |  |
| IMMT | 0,83768 |  |  |
| ELP6 | 0,8363 |  |  |
| AFAP1L2 | 0,83585 |  |  |
| AKR1B1 | 0,83554 |  |  |
| C5AR1 | 0,83366 |  |  |
| SEC24A | 0,83266 |  |  |
| C20orf197 | 0,83081 |  |  |
| C5orf15 | 0,82932 |  |  |
| IKBIP | 0,82918 |  |  |
| ZDHHC16 | 0,82551 |  |  |
| DDHD1 | 0,82454 |  |  |
| NFAT5 | 0,82415 |  |  |
| CHTF8 | 0,8233 |  |  |
| TRAPPC6B | 0,82134 |  |  |
| RNF216 | 0,82039 |  |  |
| BRWD3 | 0,82002 |  |  |
| MCPH1 | 0,81369 |  |  |
| LIMD2 | 0,8133 |  |  |
| EIF3K | 0,80644 |  |  |
| MYNN | 0,80532 |  |  |
| PCDH1 | 0,80115 |  |  |
| CRK | 0,8009 |  |  |
| PRIM2 | 0,79499 |  |  |
| ANKRD39 | 0,79307 |  |  |
| UBE4A | 0,78108 |  |  |
| E2F4 | 0,77796 |  |  |
| PPCDC | 0,7745 |  |  |
| MS4A8 | 0,76729 |  |  |
| COA5 | 0,76444 |  |  |
| C21orf2 | 0,76264 |  |  |
| EXOSC6 | 0,7625 |  |  |
| EIF4A3 | 0,76082 |  |  |
| ALG1 | 0,75811 |  |  |
| HACD3 | 0,75521 |  |  |
| CCNYL1 | 0,75033 |  |  |
| UMPS | 0,74851 |  |  |
| TSKU | 0,74687 |  |  |
| PRC1 | 0,74607 |  |  |
| RABIF | 0,74578 |  |  |
| C3orf70 | 0,74232 |  |  |
| BZW2 | 0,7394 |  |  |
| ANKLE2 | 0,73705 |  |  |
| SLC9A1 | 0,73654 |  |  |
| APPBP2 | 0,73554 |  |  |
| SMIM31 | 0,73406 |  |  |
| B3GALNT2 | 0,73313 |  |  |
| SHMT1 | 0,73123 |  |  |
| ZDHHC9 | 0,72729 |  |  |
| CAMKMT | 0,72713 |  |  |
| NDE1 | 0,72383 |  |  |
| SLC22A23 | 0,72381 |  |  |
| IRF7 | 0,72003 |  |  |
| BNIP2 | 0,7166 |  |  |
| GALC | 0,71117 |  |  |
| RPLP0P2 | 0,71109 |  |  |
| SND1 | 0,71061 |  |  |
| ARFGAP1 | 0,70965 |  |  |
| NUDT7 | 0,70731 |  |  |
| TNFAIP3 | 0,70218 |  |  |
| MCM3 | 0,70056 |  |  |
| PRPF38B | 0,69947 |  |  |
| C19orf25 | 0,69908 |  |  |
| WBP1 | 0,69812 |  |  |
| GREM1 | 0,69799 |  |  |
| OPTN | 0,6968 |  |  |
| RHBDD2 | 0,68986 |  |  |
| TIMM44 | 0,68698 |  |  |
| AGFG1 | 0,68373 |  |  |
| CAMSAP2 | 0,68372 |  |  |
| ANKRD44 | 0,68078 |  |  |
| MED28 | 0,67888 |  |  |
| UBR4 | 0,67619 |  |  |
| MED1 | 0,67577 |  |  |
| SECISBP2 | 0,67313 |  |  |
| EIF2AK3 | 0,66626 |  |  |
| AKIRIN1 | 0,66582 |  |  |
| PDZD8 | 0,66026 |  |  |
| GABPB1 | 0,65994 |  |  |
| SOCS6 | 0,65731 |  |  |
| COBL | 0,6554 |  |  |
| CD37 | 0,65463 |  |  |
| COX7A1 | 0,65137 |  |  |
| RPSAP58 | 0,65116 |  |  |
| CBFA2T2 | 0,64739 |  |  |
| LSM8 | 0,64498 |  |  |
| CSDE1 | 0,64031 |  |  |
| IFI35 | 0,6384 |  |  |
| TNPO3 | 0,62376 |  |  |
| DIS3 | 0,62312 |  |  |
| KIF2A | 0,61891 |  |  |
| GATAD1 | 0,61796 |  |  |
| EVADR | 0,61258 |  |  |
| AK4 | 0,61228 |  |  |
| TCF12 | 0,61037 |  |  |
| WWOX | 0,60773 |  |  |
| SOCS4 | 0,60454 |  |  |
| C1QA | 0,59961 |  |  |
| SERTAD3 | 0,59381 |  |  |
| SNX14 | 0,59251 |  |  |
| CCL4L2 | 0,58919 |  |  |
| DNAJB4 | 0,58498 |  |  |
| MFSD5 | 0,58437 |  |  |
| ARFRP1 | 0,58321 |  |  |
| PIGH | 0,57915 |  |  |
| FBXL15 | 0,57866 |  |  |
| TMEM184B | 0,57793 |  |  |
| ABHD12 | 0,57676 |  |  |
| USP34 | 0,57391 |  |  |
| NSD1 | 0,57044 |  |  |
| SLC12A7 | 0,56648 |  |  |
| MARVELD3 | 0,56608 |  |  |
| TATDN3 | 0,55696 |  |  |
| TNIP2 | 0,5569 |  |  |
| MATN2 | 0,53687 |  |  |
| SLC16A1 | 0,53315 |  |  |
| GK5 | 0,52595 |  |  |
| CETN3 | 0,5248 |  |  |
| CCZ1P-OR7E38P | 0,52329 |  |  |
| NEO1 | 0,51923 |  |  |
| RAPGEF1 | 0,50937 |  |  |
| CBWD5 | 0,49737 |  |  |
| NFATC2IP | 0,49616 |  |  |
| RNF41 | 0,49469 |  |  |
| MAT2A | 0,49327 |  |  |
| ATXN2 | 0,49034 |  |  |
| CHID1 | 0,48643 |  |  |
| CDC20 | 0,48621 |  |  |
| LACTB | 0,47822 |  |  |
| KIAA1468 | 0,4772 |  |  |
| SLF2 | 0,46929 |  |  |
| BTN3A2 | 0,46328 |  |  |
| WDR48 | 0,44886 |  |  |
| FURIN | 0,4446 |  |  |
| SPI1 | 0,41422 |  |  |
| F13A1 | 0,4068 |  |  |
| ELP5 | 0,40135 |  |  |
| DHX9 | 0,38697 |  |  |
| FLAD1 | 0,37101 |  |  |
| HK1 | 0,34454 |  |  |
| LIMK2 | 0,33795 |  |  |
| FAM13A | 0,337 |  |  |
| LAMB1 | 0,31693 |  |  |
| CHRNE | 0,27446 |  |  |
| NEK7 | 0,25726 |  |  |
| ALAD | 0,24075 |  |  |
| MAP4K4 | 0,20727 |  |  |
| EBP | 0,14644 |  |  |
|  |  |  |  |
