# Supplementary material for: Computer-Aided Imaging Analysis of Probe-Based Confocal Laser Endomicroscopy With Molecular Labeling and Gene Expression Identifies Markers of Response to Biological Therapy in IBD Patients: The Endo-Omics Study
Source: Inflamm Bowel Dis. 2022 Nov 15;29(9):1409–20. doi: 10.1093/ibd/izac233 (PMC10472745; doi:10.1093/ibd/izac233)
Supplement: izac233_suppl_Supplementary_Table_S3 [file izac233_suppl_supplementary_table_s3.docx]

**Supplementary Table 3:** Summary of pathway related clusters determined by DAVID Functional Annotation Clustering on the 342 DEGs. Pathways with P<0.05 are shaded in red.

| Cluster 1: | Enrichment Score: 1.3866969528786492 |  |  |  |  |  |  |  |  |  |  |  |  |
| --- | --- | --- | --- | --- | --- | --- | --- | --- | --- | --- | --- | --- | --- |
| Category | Term | Count | % | PValue | Genes | List Total | Pop Hits | Pop Total | Fold Enrichment | Bonferroni | Benjamini | FDR | General Function |
| SMART | SM00252:SH2 | 6 | 1,791044776 | 0,028305488 | YES1, SOCS6, JAK3, CRK, TNS3, SOCS4 | 168 | 103 | 10057 | 3,487170596 | 0,989889795 | 1 | 1 | Immune response |
| UP_KEYWORDS | SH2 domain | 6 | 1,791044776 | 0,029405497 | YES1, SOCS6, JAK3, CRK, TNS3, SOCS4 | 329 | 108 | 20581 | 3,475346167 | 0,999909676 | 0,278015609 | 0,260194095 |  |
| INTERPRO | IPR000980:SH2 domain | 6 | 1,791044776 | 0,046571077 | YES1, SOCS6, JAK3, CRK, TNS3, SOCS4 | 322 | 113 | 18559 | 3,060352883 | 1 | 1 | 1 |  |
| UP_SEQ_FEATURE | domain:SH2 | 5 | 1,492537313 | 0,073248163 | YES1, SOCS6, CRK, TNS3, SOCS4 | 330 | 96 | 20063 | 3,166508838 | 1 | 1 | 1 |  |
|  |  |  |  |  |  |  |  |  |  |  |  |  |  |
| Cluster 2 | Enrichment Score: 1.3299457518137687 |  |  |  |  |  |  |  |  |  |  |  |  |
| Category | Term | Count | % | PValue | Genes | List Total | Pop Hits | Pop Total | Fold Enrichment | Bonferroni | Benjamini | FDR | General Function |
| KEGG_PATHWAY | hsa04510:Focal adhesion | 11 | 3,28358209 | 0,009076966 | LAMA4, ACTN1, PXN, RAPGEF1, ILK, BRAF, LAMB1, RAC1, PPP1R12B, CRK, ARHGAP35 | 141 | 206 | 6879 | 2,605143565 | 0,849926472 | 0,472002253 | 0,472002253 | Focal Adhesion/cytoskeleton and migration |
| KEGG_PATHWAY | hsa04810:Regulation of actin cytoskeleton | 10 | 2,985074627 | 0,027090618 | ARPC3, LIMK2, ACTN1, PXN, BRAF, RAC1, PPP1R12B, CRK, ARHGAP35, SLC9A1 | 141 | 210 | 6879 | 2,323201621 | 0,996695868 | 1 | 1 |  |
| KEGG_PATHWAY | hsa04670:Leukocyte transendothelial migration | 4 | 1,194029851 | 0,416296829 | ACTN1, PXN, RAC1, ARHGAP35 | 141 | 115 | 6879 | 1,696947271 | 1 | 1 | 1 |  |
|  |  |  |  |  |  |  |  |  |  |  |  |  |  |
| Cluster 3 | Enrichment Score: 1.1283350750159258 |  |  |  |  |  |  |  |  |  |  |  |  |
| Category | Term | Count | % | PValue | Genes | List Total | Pop Hits | Pop Total | Fold Enrichment | Bonferroni | Benjamini | FDR | General Function |
| GOTERM_BP_DIRECT | GO:0000186~activation of MAPKK activity | 5 | 1,492537313 | 0,009890375 | GADD45B, RAPGEF1, BRAF, CRK, TGFBR1 | 309 | 46 | 16792 | 5,906852399 | 0,999999974 | 1 | 1 | MAP kinase signalling |
| KEGG_PATHWAY | hsa04010:MAPK signaling pathway | 9 | 2,686567164 | 0,143372372 | DUSP10, GADD45B, MAPKAPK2, BRAF, RAC1, STK4, CRK, TGFBR1, MAP4K4 | 141 | 253 | 6879 | 1,735514254 | 1 | 1 | 1 |  |
| KEGG_PATHWAY | hsa04068:FoxO signaling pathway | 5 | 1,492537313 | 0,290614018 | GADD45B, PRKAG1, BRAF, STK4, TGFBR1 | 141 | 134 | 6879 | 1,820419181 | 1 | 1 | 1 |  |
|  |  |  |  |  |  |  |  |  |  |  |  |  |  |
| Cluster 4 | Enrichment Score: 1.09131344406161 |  |  |  |  |  |  |  |  |  |  |  |  |
| Category | Term | Count | % | PValue | Genes | List Total | Pop Hits | Pop Total | Fold Enrichment | Bonferroni | Benjamini | FDR | General Function |
| GOTERM_BP_DIRECT | GO:0007264~small GTPase mediated signal transduction | 10 | 2,985074627 | 0,038141356 | MFHAS1, ARFRP1, ABR, RABIF, DOCK8, DOCK7, RAPGEF1, RAC1, HACD3, ARHGAP35 | 309 | 246 | 16792 | 2,209066751 | 1 | 1 | 1 | G protein signalling |
| GOTERM_MF_DIRECT | GO:0005085~guanyl-nucleotide exchange factor activity | 6 | 1,791044776 | 0,065064052 | ABR, RABIF, DIS3, DOCK8, DOCK7, RAPGEF1 | 309 | 118 | 16881 | 2,777850913 | 1 | 1 | 1 |  |
| UP_KEYWORDS | Guanine-nucleotide releasing factor | 5 | 1,492537313 | 0,214448213 | ABR, RABIF, DOCK8, DOCK7, RAPGEF1 | 329 | 149 | 20581 | 2,099202383 | 1 | 0,916545786 | 0,857792851 |  |
|  |  |  |  |  |  |  |  |  |  |  |  |  |  |
| Cluster 5 | Enrichment Score: 1.0299126863140196 |  |  |  |  |  |  |  |  |  |  |  |  |
| Category | Term | Count | % | PValue | Genes | List Total | Pop Hits | Pop Total | Fold Enrichment | Bonferroni | Benjamini | FDR | General Function |
| GOTERM_BP_DIRECT | GO:0000186~activation of MAPKK activity | 5 | 1,492537313 | 0,009890375 | GADD45B, RAPGEF1, BRAF, CRK, TGFBR1 | 309 | 46 | 16792 | 5,906852399 | 0,999999974 | 1 | 1 | Cell signalling |
| GOTERM_BP_DIRECT | GO:1900026~positive regulation of substrate adhesion-dependent cell spreading | 4 | 1,194029851 | 0,020468014 | BRAF, CIB1, RAC1, CRK | 309 | 32 | 16792 | 6,792880259 | 1 | 1 | 1 |  |
| KEGG_PATHWAY | hsa04910:Insulin signaling pathway | 7 | 2,089552239 | 0,061942872 | FASN, PRKAG1, RAPGEF1, BRAF, CRK, SOCS4, HK1 | 141 | 138 | 6879 | 2,47471477 | 0,999998326 | 1 | 1 |  |
| KEGG_PATHWAY | hsa05211:Renal cell carcinoma | 4 | 1,194029851 | 0,150570172 | RAPGEF1, BRAF, RAC1, CRK | 141 | 66 | 6879 | 2,956802063 | 1 | 1 | 1 |  |
| KEGG_PATHWAY | hsa04062:Chemokine signaling pathway | 7 | 2,089552239 | 0,177928512 | CXCL6, CCL4L2, PXN, BRAF, RAC1, JAK3, CRK | 141 | 186 | 6879 | 1,836078701 | 1 | 1 | 1 |  |
| KEGG_PATHWAY | hsa04722:Neurotrophin signaling pathway | 5 | 1,492537313 | 0,227828236 | MAPKAPK2, RAPGEF1, BRAF, RAC1, CRK | 141 | 120 | 6879 | 2,032801418 | 1 | 1 | 1 |  |
| KEGG_PATHWAY | hsa04015:Rap1 signaling pathway | 4 | 1,194029851 | 0,806750717 | RAPGEF1, BRAF, RAC1, CRK | 141 | 210 | 6879 | 0,929280648 | 1 | 1 | 1 |  |
|  |  |  |  |  |  |  |  |  |  |  |  |  |  |
| Cluster 6 | Enrichment Score: 1.0025008016778558 |  |  |  |  |  |  |  |  |  |  |  |  |
| Category | Term | Count | % | PValue | Genes | List Total | Pop Hits | Pop Total | Fold Enrichment | Bonferroni | Benjamini | FDR | General Function |
| GOTERM_BP_DIRECT | GO:0048013~ephrin receptor signaling pathway | 6 | 1,791044776 | 0,020952329 | YES1, ARPC3, AP2S1, AP2B1, RAC1, CRK | 309 | 86 | 16792 | 3,791375028 | 1 | 1 | 1 | Immune response |
| GOTERM_BP_DIRECT | GO:0050690~regulation of defense response to virus by virus | 3 | 0,895522388 | 0,092813962 | AP2S1, AP2B1, RAC1 | 309 | 28 | 16792 | 5,822468793 | 1 | 1 | 1 |  |
| GOTERM_BP_DIRECT | GO:0060071~Wnt signaling pathway, planar cell polarity pathway | 3 | 0,895522388 | 0,505419474 | AP2S1, AP2B1, RAC1 | 309 | 92 | 16792 | 1,77205572 | 1 | 1 | 1 |  |
|  |  |  |  |  |  |  |  |  |  |  |  |  |  |
| Cluster 7 | Enrichment Score: 0.9732630016268184 |  |  |  |  |  |  |  |  |  |  |  |  |
| Category | Term | Count | % | PValue | Genes | List Total | Pop Hits | Pop Total | Fold Enrichment | Bonferroni | Benjamini | FDR | General Function |
| SMART | SM00041:CT | 3 | 0,895522388 | 0,055216656 | GREM1, CYR61, CTGF | 168 | 23 | 10057 | 7,808229814 | 0,99988698 | 1 | 1 | Cell signalling |
| UP_SEQ_FEATURE | domain:CTCK | 3 | 0,895522388 | 0,062794206 | GREM1, CYR61, CTGF | 330 | 25 | 20063 | 7,295636364 | 1 | 1 | 1 |  |
| INTERPRO | IPR006207:Cystine knot, C-terminal | 3 | 0,895522388 | 0,073842431 | GREM1, CYR61, CTGF | 322 | 26 | 18559 | 6,650382226 | 1 | 1 | 1 |  |
| GOTERM_BP_DIRECT | GO:0007267~cell-cell signaling | 6 | 1,791044776 | 0,4996344 | GREM1, CXCL6, C1QA, PCDH1, CYR61, CTGF | 309 | 254 | 16792 | 1,283693907 | 1 | 1 | 1 |  |
|  |  |  |  |  |  |  |  |  |  |  |  |  |  |
| Cluster 8 | Enrichment Score: 0.8463192896720094 |  |  |  |  |  |  |  |  |  |  |  |  |
| Category | Term | Count | % | PValue | Genes | List Total | Pop Hits | Pop Total | Fold Enrichment | Bonferroni | Benjamini | FDR | General Function |
| GOTERM_BP_DIRECT | GO:0048013~ephrin receptor signaling pathway | 6 | 1,791044776 | 0,020952329 | YES1, ARPC3, AP2S1, AP2B1, RAC1, CRK | 309 | 86 | 16792 | 3,791375028 | 1 | 1 | 1 | Immune response |
| KEGG_PATHWAY | hsa05100:Bacterial invasion of epithelial cells | 5 | 1,492537313 | 0,074000393 | ARPC3, PXN, ILK, RAC1, CRK | 141 | 78 | 6879 | 3,127386798 | 0,999999886 | 1 | 1 |  |
| KEGG_PATHWAY | hsa04666:Fc gamma R-mediated phagocytosis | 4 | 1,194029851 | 0,243981324 | ARPC3, LIMK2, RAC1, CRK | 141 | 84 | 6879 | 2,323201621 | 1 | 1 | 1 |  |
| KEGG_PATHWAY | hsa05131:Shigellosis | 3 | 0,895522388 | 0,375635713 | ARPC3, RAC1, CRK | 141 | 64 | 6879 | 2,286901596 | 1 | 1 | 1 |  |
| GOTERM_BP_DIRECT | GO:0038096~Fc-gamma receptor signaling pathway involved in phagocytosis | 4 | 1,194029851 | 0,412870406 | YES1, ARPC3, RAC1, CRK | 309 | 127 | 16792 | 1,711591876 | 1 | 1 | 1 |  |
|  |  |  |  |  |  |  |  |  |  |  |  |  |  |
| Cluster 9 | Enrichment Score: 0.7827902121250724 |  |  |  |  |  |  |  |  |  |  |  |  |
| Category | Term | Count | % | PValue | Genes | List Total | Pop Hits | Pop Total | Fold Enrichment | Bonferroni | Benjamini | FDR | General Function |
| GOTERM_BP_DIRECT | GO:0019886~antigen processing and presentation of exogenous peptide antigen via MHC class II | 5 | 1,492537313 | 0,088976605 | SEC24A, KIF2A, AP2S1, AP2B1, HLA-DQB2 | 309 | 92 | 16792 | 2,9534262 | 1 | 1 | 1 | Immune response |
| GOTERM_CC_DIRECT | GO:0030669~clathrin-coated endocytic vesicle membrane | 3 | 0,895522388 | 0,158592745 | AP2S1, AP2B1, HLA-DQB2 | 317 | 41 | 18224 | 4,206509194 | 1 | 1 | 0,969135802 |  |
| GOTERM_CC_DIRECT | GO:0030666~endocytic vesicle membrane | 3 | 0,895522388 | 0,317738774 | AP2S1, AP2B1, HLA-DQB2 | 317 | 66 | 18224 | 2,6131345 | 1 | 1 | 0,969135802 |  |
|  |  |  |  |  |  |  |  |  |  |  |  |  |  |
| Cluster 10 | Enrichment Score: 0.7202623019463578 |  |  |  |  |  |  |  |  |  |  |  |  |
| Category | Term | Count | % | PValue | Genes | List Total | Pop Hits | Pop Total | Fold Enrichment | Bonferroni | Benjamini | FDR | General Function |
| GOTERM_BP_DIRECT | GO:0051496~positive regulation of stress fiber assembly | 4 | 1,194029851 | 0,041477365 | BRAF, RAC1, TGFBR1, CTGF | 309 | 42 | 16792 | 5,175527816 | 1 | 1 | 1 | Cancer |
| BIOCARTA | h_mapkPathway:MAPKinase Signaling Pathway | 5 | 1,492537313 | 0,229394274 | MAPKAPK2, BRAF, RAC1, TGFBR1, MAP4K4 | 47 | 87 | 1625 | 1,987038396 | 1 | 1 | 1 |  |
| KEGG_PATHWAY | hsa05210:Colorectal cancer | 3 | 0,895522388 | 0,360939256 | BRAF, RAC1, TGFBR1 | 141 | 62 | 6879 | 2,360672615 | 1 | 1 | 1 |  |
| KEGG_PATHWAY | hsa05212:Pancreatic cancer | 3 | 0,895522388 | 0,382933133 | BRAF, RAC1, TGFBR1 | 141 | 65 | 6879 | 2,251718494 | 1 | 1 | 1 |  |
|  |  |  |  |  |  |  |  |  |  |  |  |  |  |
| Cluster 11 | Enrichment Score: 0.7197595106965798 |  |  |  |  |  |  |  |  |  |  |  |  |
| Category | Term | Count | % | PValue | Genes | List Total | Pop Hits | Pop Total | Fold Enrichment | Bonferroni | Benjamini | FDR | General Function |
| INTERPRO | IPR013781:Glycoside hydrolase, catalytic domain | 3 | 0,895522388 | 0,134021157 | CHID1, GALC, SLC3A1 | 322 | 37 | 18559 | 4,673241565 | 1 | 1 | 1 | Carbohdrate metabolism |
| GOTERM_BP_DIRECT | GO:0005975~carbohydrate metabolic process | 6 | 1,791044776 | 0,215706114 | CHID1, GALC, GK5, RPE, AKR1B1, SLC3A1 | 309 | 174 | 16792 | 1,873898002 | 1 | 1 | 1 |  |
| INTERPRO | IPR017853:Glycoside hydrolase, superfamily | 3 | 0,895522388 | 0,239709724 | CHID1, GALC, SLC3A1 | 322 | 54 | 18559 | 3,202035887 | 1 | 1 | 1 |  |
|  |  |  |  |  |  |  |  |  |  |  |  |  |  |
| Cluster 12 | Enrichment Score: 0.5250149374196633 |  |  |  |  |  |  |  |  |  |  |  |  |
| Category | Term | Count | % | PValue | Genes | List Total | Pop Hits | Pop Total | Fold Enrichment | Bonferroni | Benjamini | FDR | General Function |
| UP_KEYWORDS | Cholesterol metabolism | 3 | 0,895522388 | 0,230262088 | MBTPS1, EBP, PMVK | 329 | 57 | 20581 | 3,292433211 | 1 | 0,933010019 | 0,873201685 | Sterol Metabolism |
| UP_KEYWORDS | Sterol metabolism | 3 | 0,895522388 | 0,289382151 | MBTPS1, EBP, PMVK | 329 | 67 | 20581 | 2,801025269 | 1 | 1 | 0,938906752 |  |
| UP_KEYWORDS | Steroid metabolism | 3 | 0,895522388 | 0,399264786 | MBTPS1, EBP, PMVK | 329 | 86 | 20581 | 2,182194105 | 1 | 1 | 0,938906752 |  |
|  |  |  |  |  |  |  |  |  |  |  |  |  |  |
| Cluster 13 | Enrichment Score: 0.39886314022978225 |  |  |  |  |  |  |  |  |  |  |  |  |
| Category | Term | Count | % | PValue | Genes | List Total | Pop Hits | Pop Total | Fold Enrichment | Bonferroni | Benjamini | FDR | General Function |
| GOTERM_CC_DIRECT | GO:0031012~extracellular matrix | 9 | 2,686567164 | 0,144121599 | ADAMTS4, LAMA4, ILK, CRIP2, LAMB1, EMILIN1, RAC1, CYR61, MATN2 | 317 | 296 | 18224 | 1,747975104 | 1 | 1 | 0,969135802 | Extracellular Matrix |
| UP_KEYWORDS | Cell adhesion | 10 | 2,985074627 | 0,357332568 | CXADR, LAMA4, PXN, CIB1, LAMB1, EMILIN1, PCDH1, NEO1, CYR61, CTGF | 329 | 479 | 20581 | 1,305975595 | 1 | 1 | 0,938906752 |  |
| GOTERM_BP_DIRECT | GO:0030198~extracellular matrix organization | 5 | 1,492537313 | 0,485081112 | LAMA4, FURIN, LAMB1, EMILIN1, CYR61 | 309 | 196 | 16792 | 1,386302094 | 1 | 1 | 1 |  |
| UP_KEYWORDS | Extracellular matrix | 5 | 1,492537313 | 0,591070921 | ADAMTS4, LAMA4, LAMB1, EMILIN1, CTGF | 329 | 258 | 20581 | 1,212330058 | 1 | 1 | 0,938906752 |  |
| GOTERM_CC_DIRECT | GO:0005578~proteinaceous extracellular matrix | 5 | 1,492537313 | 0,68616517 | ADAMTS4, EMILIN1, CYR61, CTGF, MATN2 | 317 | 268 | 18224 | 1,072555205 | 1 | 1 | 0,969135802 |  |
|  |  |  |  |  |  |  |  |  |  |  |  |  |  |
| Cluster 14 | Enrichment Score: 0.39712485961630123 |  |  |  |  |  |  |  |  |  |  |  |  |
| Category | Term | Count | % | PValue | Genes | List Total | Pop Hits | Pop Total | Fold Enrichment | Bonferroni | Benjamini | FDR | General Function |
| UP_KEYWORDS | Chemotaxis | 4 | 1,194029851 | 0,201533421 | CXCL6, CCL4L2, C5AR1, CYR61 | 329 | 97 | 20581 | 2,579638392 | 1 | 0,898263247 | 0,84068227 | Immune response |
| GOTERM_BP_DIRECT | GO:0060326~cell chemotaxis | 3 | 0,895522388 | 0,335325263 | CXCL6, CCL4L2, C5AR1 | 309 | 65 | 16792 | 2,508140403 | 1 | 1 | 1 |  |
| GOTERM_BP_DIRECT | GO:0006955~immune response | 5 | 1,492537313 | 0,952383956 | CXCL6, CCL4L2, C5AR1, TCF12, HLA-DQB2 | 309 | 421 | 16792 | 0,6454043 | 1 | 1 | 1 |  |
|  |  |  |  |  |  |  |  |  |  |  |  |  |  |
| Cluster 15 | Enrichment Score: 0.3640034692570718 |  |  |  |  |  |  |  |  |  |  |  |  |
| Category | Term | Count | % | PValue | Genes | List Total | Pop Hits | Pop Total | Fold Enrichment | Bonferroni | Benjamini | FDR | General Function |
| GOTERM_BP_DIRECT | GO:0098609~cell-cell adhesion | 7 | 2,089552239 | 0,378847148 | PUF60, YWHAB, FASN, RPL14, BZW2, SND1, EIF2A | 309 | 271 | 16792 | 1,403694814 | 1 | 1 | 1 | Cell Adhesion |
| GOTERM_MF_DIRECT | GO:0098641~cadherin binding involved in cell-cell adhesion | 7 | 2,089552239 | 0,435792408 | PUF60, YWHAB, FASN, RPL14, BZW2, SND1, EIF2A | 309 | 290 | 16881 | 1,318680951 | 1 | 1 | 1 |  |
| GOTERM_CC_DIRECT | GO:0005913~cell-cell adherens junction | 7 | 2,089552239 | 0,490056225 | PUF60, YWHAB, FASN, RPL14, BZW2, SND1, EIF2A | 317 | 323 | 18224 | 1,245890752 | 1 | 1 | 0,969135802 |  |
|  |  |  |  |  |  |  |  |  |  |  |  |  |  |
| Cluster 16 | Enrichment Score: 0.07092206678854032 |  |  |  |  |  |  |  |  |  |  |  |  |
| Category | Term | Count | % | PValue | Genes | List Total | Pop Hits | Pop Total | Fold Enrichment | Bonferroni | Benjamini | FDR | General Function |
| UP_KEYWORDS | Innate immunity | 4 | 1,194029851 | 0,788547179 | CHID1, C1QA, IRF7, JAK3 | 329 | 261 | 20581 | 0,958716184 | 1 | 1 | 0,938906752 | Immune response |
| UP_KEYWORDS | Immunity | 7 | 2,089552239 | 0,811434093 | CHID1, C1QA, ERAP1, IRF7, BTN3A2, JAK3, HLA-DQB2 | 329 | 500 | 20581 | 0,875787234 | 1 | 1 | 0,938906752 |  |
| GOTERM_BP_DIRECT | GO:0045087~innate immune response | 5 | 1,492537313 | 0,957531051 | CHID1, C1QA, YES1, IRF7, JAK3 | 309 | 430 | 16792 | 0,631895838 | 1 | 1 | 1 |  |
|  |  |  |  |  |  |  |  |  |  |  |  |  |  |
|  |  |  |  |  |  |  |  |  |  |  |  |  |  |
