# Supplementary material for: Computer-Aided Imaging Analysis of Probe-Based Confocal Laser Endomicroscopy With Molecular Labeling and Gene Expression Identifies Markers of Response to Biological Therapy in IBD Patients: The Endo-Omics Study
Source: Inflamm Bowel Dis. 2022 Nov 15;29(9):1409–20. doi: 10.1093/ibd/izac233 (PMC10472745; doi:10.1093/ibd/izac233)
Supplement: izac233_suppl_Supplementary_Table_S4 [file izac233_suppl_supplementary_table_s4.docx]

**Supplementary Table 4:** A summary of the AUC scores achieved for enriched genes with VIP>1 in the UC/CD and validation cohorts. Genes with AUC>0.7 for both studies are shaded.

| **Genes** | **UC/CD**  **AUC score** | **Validation AUC score** |
| --- | --- | --- |
| **ACTN1** | **0.895** | **0.866** |
| ADAMTS4 | 0.711 |  |
| AP2B1 | 0.789 | 0.504 |
| AP2S1 | 0.789 | 0.505 |
| ARHGAP35 | 0.763 | 0.589 |
| ARPC3 | 0.947 | 0.570 |
| CIB1 | 0.711 | 0.599 |
| **CRIP2** | **0.711** | **0.805** |
| CTGF | 0.728 |  |
| CXADR | 0.732 | 0.663 |
| **CXCL6** | **0.754** | **0.893** |
| CYR61 | 0.772 |  |
| DOCK7 | 0.737 |  |
| DOCK8 | 0.781 |  |
| EIF2A | 0.763 |  |
| **EMILIN1** | **0.781** | **0.739** |
| ERAP1 | 0.754 | 0.627 |
| FASN | 0.754 | 0.605 |
| **GADD45B** | **0.711** | **0.803** |
| HLA-DQB2 | 0.702 | 0.509 |
| ILK | 0.768 | 0.576 |
| JAK3 | 0.732 | 0.546 |
| **LAMA4** | **0.719** | **0.802** |
| **MAPKAPK2** | **0.697** | **0.762** |
| MBTPS1 | 0.711 | 0.568 |
| PMVK | 0.868 | 0.554 |
| PPP1R12B | 0.711 | 0.544 |
| PUF60 | 0.825 | 0.588 |
| PXN | 0.719 | 0.556 |
| RAC1 | 0.930 | 0.542 |
| RPE | 0.728 | 0.543 |
| RPL14 | 0.816 | 0.509 |
| SLC3A1 | 0.728 | 0.649 |
| STK4 | 0.798 | 0.659 |
| TGFBR1 | 0.772 | 0.678 |
| YES1 | 0.768 | 0.619 |
| YWHAB | 0.719 | 0.531 |
